# Serine hydroxymethyltransferase 2 knockdown induces apoptosis in ccRCC by causing lysosomal membrane permeabilization via metabolic reprogramming

**DOI:** 10.1038/s41419-023-05677-4

**Published:** 2023-02-20

**Authors:** Zhangnan Liu, Mengzhen Fan, Junqing Hou, Sijing Pan, Yanxin Xu, Hailong Zhang, Chen Liu, Xiangjun Hao, Xia Li, Huijuan Wang

**Affiliations:** 1 Joint National Laboratory for Antibody Drug Engineering, The First Affiliated Hospital of Henan University, School of Medicine, Henan University, Kaifeng, China; 2 Biotherapy Center, The First Affiliated Hospital of Zhengzhou University, Zhengzhou, China; 3 Henan Province Prostate Disease Prevention and Diagnosis Engineering Research Center, Henan University, Kaifeng, China; 4 Institute of Translational Medicine, School of Medicine, Henan University, Kaifeng, China

**Keywords:** Cancer metabolism, Cancer metabolism

## Abstract

Serine hydroxymethyltransferase 2 (SHMT2) plays an important role in converting serine to glycine and supplying carbon to one-carbon metabolism to sustain cancer cell proliferation. However, the expression, function, and underlying mechanisms of SHMT2 in clear cell renal cell carcinoma (ccRCC) remain largely unknown. In this study, we demonstrated that SHMT2 was upregulated in ccRCC tissues compared with controls and associated with patient survival. SHMT2 knockdown inhibited proliferation, migration, and invasion in ccRCC cells. Overexpression of SHMT2 promoted tumor progression. Mechanistically, SHMT2 depletion disrupted one-carbon metabolism, increased reactive oxygen species (ROS) levels, and decreased ATP levels via metabolic reprogramming, which destroyed cell homeostasis. The SHMT2 knockdown-induced stress activated autophagy. A mass of autophagosomes fused with lysosomes, resulting in lysosomal membrane permeabilization (LMP) and leakage of lysosomal contents into the cytoplasm, which eventually led to apoptosis. Our work reveals that SHMT2 functions as an oncogenic gene to promote ccRCC progression. SHMT2 depletion induces apoptosis by causing LMP through excessive activation of the autophagy-lysosome pathway via metabolic reprogramming.

## Introduction

Renal cell carcinoma (RCC) accounts for 2-3% of all adult malignancies, of which ccRCC is the most common subtype of RCC [1]. ccRCC is an aggressive cancer originating from the proximal tubular epithelium and the metastatic form of it is associated with high mortality [2]. With the emergence of various targeted drugs and new immunotherapy drugs, the treatment and prognosis of ccRCC have greatly improved, but therapeutic effects are not yet satisfactory [3]. Therefore, the underlying molecular mechanisms of ccRCC neoplasia and development must be elucidated to identify new therapeutic targets and develop new treatment strategies. The discovery of the Warburg effect in 1956 and modern studies have demonstrated that metabolic reprogramming is one significant hallmark of cancer [4, 5]. ccRCC is also described as a “cell metabolic disease”. Elucidation of the metabolic alterations in tumor cells provides new insights into the molecular mechanisms underlying ccRCC. Therefore, the dysregulated metabolic pathways may serve as targets for more effective ccRCC treatment.

Serine, a nonessential amino acid, can be taken up from the diets or de novo synthesized by the serine synthesis pathway (SSP) [6]. Serine, the major one-carbon donor, is catabolized to glycine by SHMTs, providing a carbon for one-carbon metabolism, which consists of two interconnected cycles: the folate cycle and the methionine cycle. The outputs of one-carbon metabolism include the biosynthesis of nucleotides, proteins, and lipids, which are required for vital functions such as maintaining the cell redox balance, and providing the substrate for methylation modifications [7]. SHMT has two isozymes: SHMT1 (cytoplasm) and SHMT2 (mitochondria). SHMT2 is upregulated in various cancers and supports tumor cell proliferation [8–10]. A large-scale comparative oncogenomics study of 392 primary human cancers showed that overexpression of SHMT2 was necessary for tumor cell survival and associated with poor prognosis in human cancer [10]. Gene set enrichment analysis confirmed the SHMT2 significance as a prognostic biomarker for ER-negative breast cancer patients. And the high expression of SHMT2 was significantly associated with breast cancer tumor aggressiveness and poor survival [11]. Some studies have even reported that tumor cells required SHMT2 to support tumor growth by maintaining the redox balance and cell survival [12, 13]. Transcriptional upregulation of SHMT2 by NRF2 supported the production of glutathione (GSH) and nucleotides, which conferred poor prognosis in human NSCLC and was linked to clinical aggressiveness [14]. In addition, downregulation of SHMT2 suppressed tumorigenesis in human hepatocellular carcinoma [15]. The above studies showed that SHMT2 played an important role in the development of tumors. However, there are few reports on the role of SHMT2 in renal cancer. Only one study based on statistical analysis of a database pointed out the clinical significance of SHMT2 in kidney cancer and provided a potential therapeutic target [16]. However, its biological function as well as underlying mechanism in kidney cancer are still unclear.

Therefore, in the present study, our purpose was to explore the effects of SHMT2 on ccRCC cell progression and the underlying mechanism. Our results indicated SHMT2 was aberrantly upregulated in ccRCC tissue and negatively associated with patient survival. SHMT2 sustained ccRCC cell progression. Further, SHMT2 depletion induced LMP and apoptosis via the autophagy-lysosome pathway. Finally, we explored the underlying mechanism of SHMT2 depletion inhibiting the progression of ccRCC through metabolic reprogramming. Taken together, our results confirmed that SHMT2 plays an important role in promoting ccRCC progression through the autophagy-lysosome pathway by metabolic reprogramming.

## Results

### Mitochondrial isoform SHMT2 was aberrantly upregulated in ccRCC tissues and associated with patient survival

**Fig. 1:**
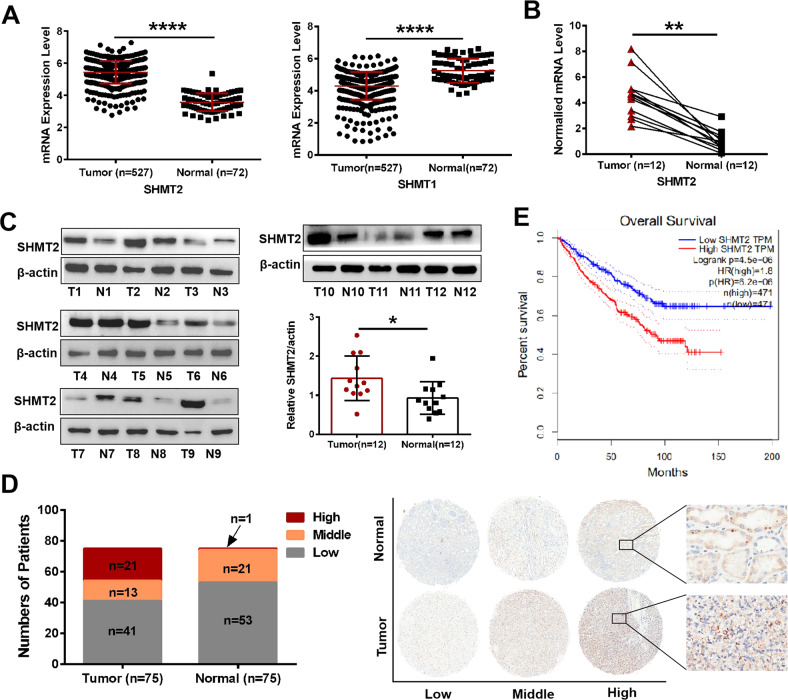
SHMT2 was aberrantly upregulated in ccRCC tissues and associated with patient survival. **A** Relative expression of SHMT2 and SHMT1 in ccRCC specimens and controls based on the TCGA database. **B** qRT-PCR analysis was performed to evaluate the *SHMT2* mRNA levels in 12 pairs of ccRCC tissues and controls. **C** Western blot analysis was performed to evaluate the SHMT2 protein levels in 12 pairs of ccRCC tissues and controls. β-actin expression was detected as a loading control. T meaned tumors and N meaned paired controls. **D** TMA IHC scores showing that high, middle and low SHMT2 expression were observed in 21, 13, and 41 of the 75 tumor specimens. The corresponding numbers were 1, 21, and 53 in the 75 paired control tissues (left). Representative IHC staining of SHMT2 protein in human ccRCC tissues (right). **E** Overall survival curves of ccRCC patients with different SHMT2 expression levels based on the GEPIA database. *P < 0.05, **P < 0.01, ****P < 0.0001.

SHMTs comprised two isozymes of SHMT2 and SHMT1 in cells, we performed bioinformatics analysis based on The Cancer Genome Atlas (TCGA) database and found that SHMT2, but not SHMT1, was overexpressed in cancer specimens, with an average 1.522-fold increase compared with controls (Fig. 1A). To investigate the aberrant expression of SHMT2, we first analyzed the mRNA and protein expression levels in 12 pairs of ccRCC specimens using qRT-PCR (Fig. 1B) and western blot (Fig. 1C), respectively, which showed that SHMT2 was markedly upregulated in ccRCC tissues. To verify the protein expression of SHMT2 in ccRCC, we further performed immunohistochemical (IHC) staining in 75 pairs of tissue samples from tissue microarrays (TMAs). The cohort was divided into three subgroups with high, middle and low SHMT2 expression based on the IHC score. The ccRCC tissues had significantly higher IHC scores compared with adjacent normal tissues (Fig. 1D). Furthermore, the ccRCC patients’ overall survival was negatively correlated with the SHMT2 expression levels ((Fig. 1E). Patients with low SHMT2 expression had a significantly better survival rate than those with high SHMT2 expression. Taken together, these data suggest that the mRNA and protein expression levels of SHMT2 are elevated in ccRCC tissues and may be associated with patient survival.

### SHMT2 sustained ccRCC proliferation and metastasis in vitro and in vivo

We profiled SHMT2 expression in a panel of ccRCC cell lines (Fig. S1A). Among them, SHMT2 showed higher levels in ACHN and A498 cells and lower levels in Caki-2 and 786-O cells. Therefore, we first knocked down SHMT2 in ACHN and A498 cells. The knockdown efficiency was confirmed (Fig. S1B). A CCK-8 assay showed that SHMT2 knockdown decreased the cell proliferation rate (Fig. S1C). And the colony numbers and sizes were reduced after SHMT2 knockdown (Fig. S1D). In addition, transwell assays demonstrated that SHMT2 knockdown also inhibited the migration and invasion abilities of ACHN and A498 cells (Fig. S1E, F).

**Fig. 2:**
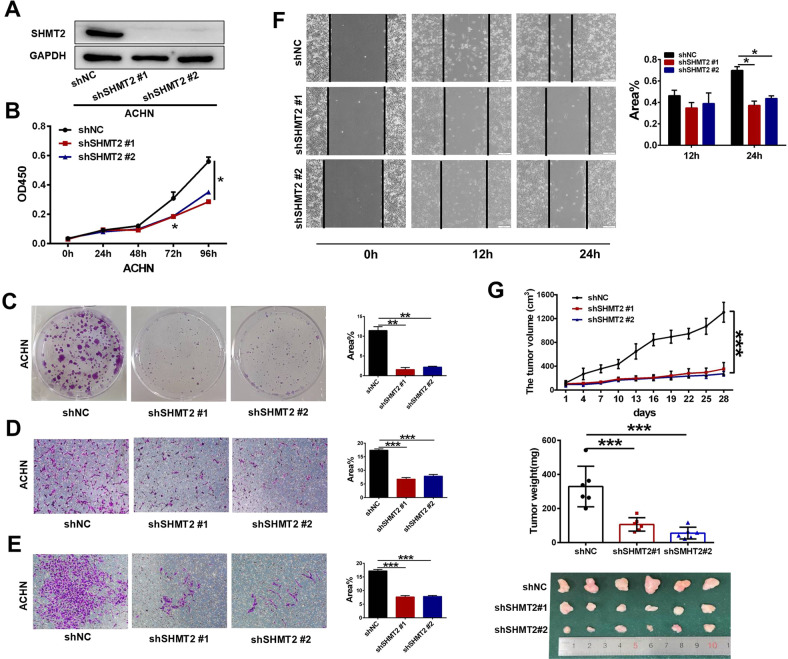
Effects of stable SHMT2 knockdown on proliferation and metastasis in ccRCC cells. Lentivirus encoding scramble control shNC or targeted shSHMT2 vectors were infected into ACHN cells. **A** Western blot analysis was performed to evaluate SHMT2 protein levels in negative control (shNC) or shSHMT2 #1/#2 cells. **B** Cell proliferation assay of ACHN cells stably transfected with shNC or shSHMT2 #1/#2. Error bars represent SD (*n* = 3). **C** Colony formation assays of ACHN cells stably transfected with shNC or shSHMT2 #1/#2 for 10 days. Each experiment was performed in triplicate. **D, E** Cell migration (**D**) and invasion (**E**) assays of ACHN cells stably transfected with shNC or shSHMT2 #1/#2. **F** Would healing assay of ACHN cells stably transfected with shNC or shSHMT2 #1/#2. **G** The tumor volume, weight, and images of shNC and shSHMT2 #1/#2 ACHN xenograft in nude mice. Tumor sizes were measured every 3 days for 4 weeks (top panel). Tumor weights were measured after dissection (middle panel). Tumor images were taken after dissection (bottom panel). Data were represented as mean ± SD. *P < 0.05, **P < 0.01, ***P < 0.001.

To further explore the function of SHMT2, we successfully created stable SHMT2 knockdown ACHN cells by transfection of lentivirus with SHMT2 shRNA (Fig. 2A). Similar to the siRNA experiments, the cell proliferation (Fig. 2B), colony formation (Fig. 2C), migration (Fig. 2D), invasion (Fig. 2E) and wound healing (Fig. 2F) were significantly suppressed after SHMT2 knockdown. Following these in vitro outcomes, we examined the cell tumorigenicity in vivo by inoculating SHMT2 knockdown ACHN cells into nude mice. As shown in Fig. 2G, the tumors derived from ACHN shSHMT2 cells exhibited a remarkably slower growth rate, lighter tumor weight, and smaller tumor size.

**Fig. 3:**
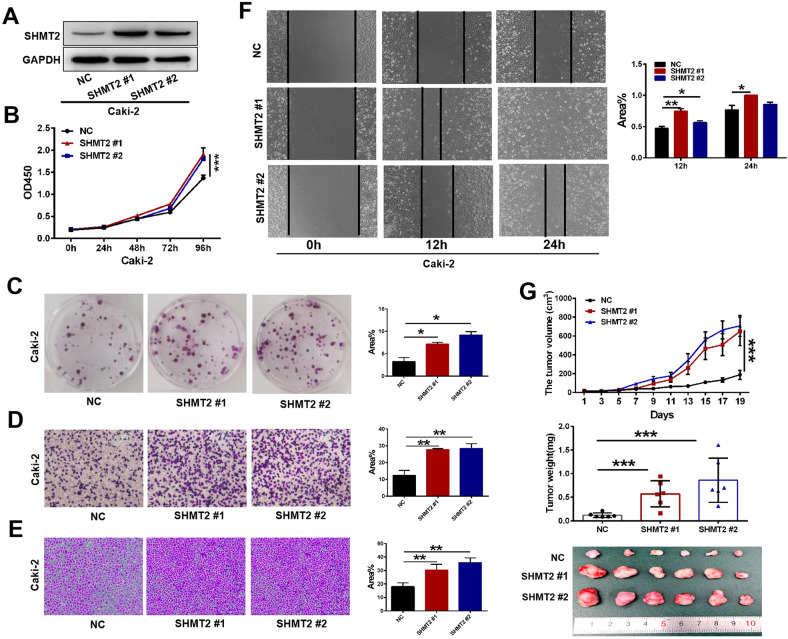
Effects of SHMT2 overexpression on proliferation and metastasis in ccRCC cells. Lentivirus encoding empty vector or SHMT2 vector were infected into Caki-2 cells. **A** Western blot analysis was performed to evaluate the SHMT2 protein levels in empty vector control (NC) or SHMT2 #1/#2 overexpression cells. **B** Cell proliferation assay of Caki-2 cells stable transfected with NC or SHMT2 #1/#2. Error bars represent SD (*n* = 3). **C** Colony formation assays of Caki-2 cells stable transfected with NC or SHMT2 #1/#2 for 10 days. **D**, **E** Cell migration (**D**) and invasion (**E**) assays of Caki-2 cells stable transfected with NC or SHMT2 #1/#2. **F** Would healing assay of Caki-2 cells stable transfected with NC or SHMT2 #1/#2. **G** The tumor volume, weight, and images of NC and SHMT2 #1/#2 Caki-2 xenograft in nude mice. Tumor sizes were measured every 2 days for total 19 days (top panel). Tumor weights were measured after dissection (middle panel). Tumor images were taken after dissection (bottom panel). Data were represented as mean ± SD. *P < 0.05, **P < 0.01, ***P < 0.001.

To assess whether SHMT2 promoted ccRCC progression, we overexpressed SHMT2 in Caki-2 cells with a lentivirus expression vector (Fig. 3A). As expected, SHMT2 overexpression significantly increased cell proliferation (Fig. 3B) and colony formation (Fig. 3C). Additionally, migration, matrigel invasion, and wound healing were enhanced after SHMT2 overexpression (Fig. 3D–F). The xenograft experiment demonstrated that SHMT2 overexpression also increased Caki-2 cell tumorigenicity in vivo (Fig. 3G). To avoid variability between cell lines, we also knocked down and overexpressed SHMT2 in A498 and 786-O cells, respectively. Consistent with our previous results, SHMT2 knockdown inhibited cell proliferation and metastasis (Fig. S2A–F), while SHMT2 overexpression promoted cell proliferation and metastasis (Fig. S2G–L). Collectively, our results suggest that SHMT2 sustains ccRCC proliferation and metastasis in vitro and in vivo.

### SHMT2 knockdown induced the autophagy-lysosome pathway in ccRCC cells

**Fig. 4:**
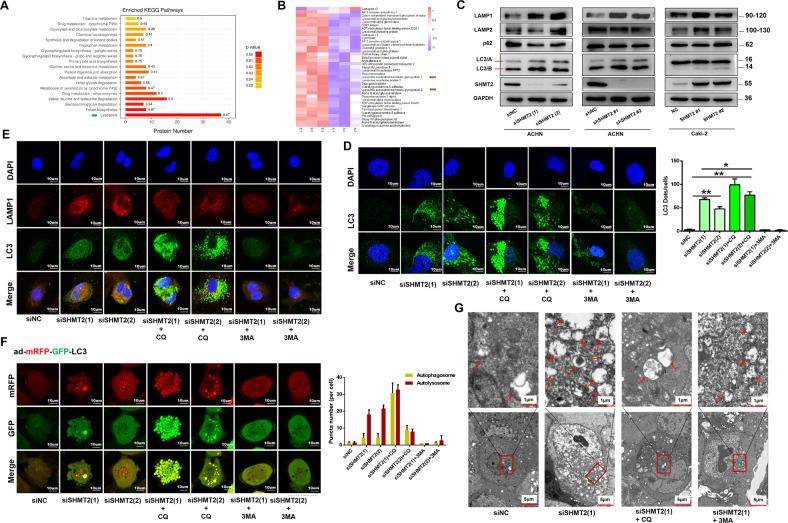
SHMT2 knockdown in ccRCC cells induced the autophagy-lysosome pathway and affected autophagic flux. **A** Top enriched KEGG pathways based on the differentially expressed proteins between shSHMT2 and shNC ACHN cells. The abscissa represented the number of differentially expressed proteins in each pathway. The color of the bar chart indicated the significance of the enriched KEGG pathways. **B** Heat-map of 37 differentially expressed proteins involved in the lysosome pathway. Red represented significantly upregulated proteins and blue represented significantly downregulated proteins after SHMT2 knockdown. Red arrows indicated the LAMP1 and LAMP2. A represented shNC, and B represented shSHMT2. **C** The levels of autophagy-lysosome-related proteins after knockdown or overexpression of SHMT2 in ccRCC cells. **D** Immunofluoresence analysis of LC3 (green) in ACHN cells after SHMT2 knockdown alone or in combination with CQ (20uM) or 3-MA (5 mM) for 48 h. Nuclei were stained with DAPI. **E** Double immunofluoresence analysis of the co-localization of LC3 (green) and LAMP1 (red) in ACHN cells after SHMT2 knockdown alone or in combination with CQ (20uM) or 3-MA (5 mM) for 48 h. Nuclei were stained with DAPI. **F** ACHN cells were transfected with adenovirus harboring mRFP-GFP-LC3 (green and red puncta indicate GFP and mRFP, respectively) to follow autophagic flux, after SHMT2 knockdown following treatment with CQ (20uM) or 3-MA (5 mM). Nuclei were stained with DAPI (left). Quantitative analysis of autophagosomes (yellow dots) and autolysosomes (red dots) (right). **G** TEM analysis revealed that autolysome formation was induced after SHMT2 knockdown in ACHN cells. Red arrows indicated autolysosomes or autophagosomes. The scale bars in the original image and the enlarged image represented 5 μm and 1 μm, respectively. *P < 0.05, **P < 0.01.

We performed cell proteomic analysis using LC-MS/MS after SHMT2 knockdown in ACHN cells. A total of 676 differentially expressed proteins were upregulated in shSHMT2 cells, while 510 proteins were downregulated. The top enriched KEGG pathways among these differentially expressed proteins were shown in Fig. 4A. Among them, the significance of the lysosome pathway was the highest (green arrow), with 37 differentially expressed proteins (Fig. 4B), of which lysosome-associated membrane protein 1 (LAMP1) and LAMP2 were upregulated (red arrows). LAMP1 expression was indeed significantly increased after SHMT2 knockdown (Fig. 4C), indicating that the lysosome pathway was enhanced. LC3 is a hallmark protein of autophagy. With the activation of autophagy, LC3-I is recruited to autophagosomes and then converted to LC3-II. Our results showed that the LC3-II/LC3-I ratio was strongly increased after SHMT2 knockdown, which indicated the autophagy was activated.

Immunofluorescence staining results also showed that LC3 puncta were markedly increased after SHMT2 knockdown (Fig. 4D). Generally, autophagosome degradation depends on its fusion with acidic lysosomes. In the following experiments, CQ was used to block autolysosome formation and 3-MA was specifically used to inhibit autophagy by preventing the formation of acidic vacuoles. CQ treatment significantly enhanced LC3 puncta, while 3-MA treatment obviously reduced LC3 puncta in siSHMT2 cells (Fig. 4D). SHMT2 knockdown increased the levels of co-localization with LC3 and LAMP1 (Fig. 4E). CQ treatment only increased green LC3 puncta and 3-MA treatment inhibited both the green LC3 puncta and red LAMP1 puncta and their co-localization, which suggested the autophagy-lysosome pathway may be activated after SHMT2 knockdown. Furthermore, we also infected SHMT2-knockdown ACHN cells with mRFP-GFP-LC3 adenovirus to monitor autophagic flux. Correspondingly, both yellow (mRFP and GFP) and red (mRFP only) puncta will increase as autophagic flux increases. Once blocking the autophagosome fusion with the lysosomes (i.e., CQ treatment) or inhibiting lysosomal degradation would only enhance the number of yellow dots. As shown in Fig. 4F, both yellow and red puncta were increased in siSHMT2 cells due to the increased autophagic flux. On the contrary, blockage of fusion with CQ only resulted in increased yellow puncta. In addition, we performed transmission electron microscopy (TEM) to detect autophagosomes and autolysosomes, and found that the number of autophagosomes and autolysosomes were markedly increased after SHMT2 knockdown (Fig. 4G).

To avoid variability between cell lines, we also detected the autophagy-lysosome pathway in A-498 cells. Consistent with the results in ACHN cells, the co-localization with LC3 and LAMP1, and the autophagic flux were both increased after SHMT2 knockdown, while decreased following treatment with CQ or 3-MA (Fig. S3A, B). Collectively, these results suggest that SHMT2 knockdown actually overactivates autophagy-lysosome pathway in ccRCC cells.

### Inhibition of autophagy decreased SHMT2 knockdown induced lysosomal membrane permeabilization and cell apoptosis in ccRCC cells

**Fig. 5:**
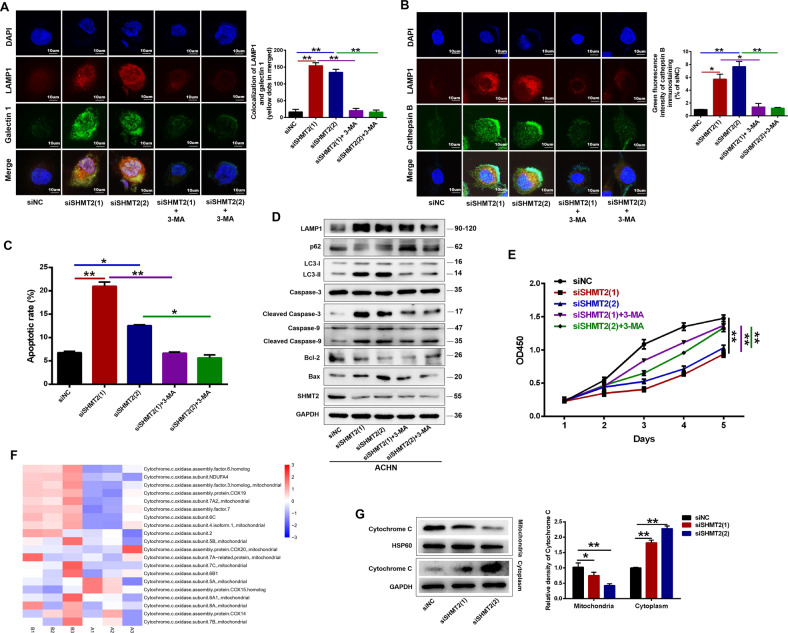
Inhibition of autophagy reduced SHMT2 knockdown-induced LMP and apoptosis in ccRCC cells. **A** Double immunofluorescence analysis of the co-localization of galectin-1 (green) and LAMP1 (red) in ACHN cells after SHMT2 knockdown alone or in combination with 3-MA (5 mM) for 48 h. Nuclei were stained with DAPI. Green Galectin punctea indicated compromised lysosomes (left). Co-localization of galectin 1 and LAMP1 was quantified using NIH Image J (right). **B** Double immunofluorescence staining of cathepsin B (green) and LAMP1 (red) in ACHN cells after SHMT2 knockdown following treatment with 3-MA (5 mM). Nuclei were stained with DAPI (left). Quantification of green fluorescence intensity of cathepsin B immunostaining (right). **C** Quantitative FACS apoptosis results in ACHN cells after SHMT2 knockdown alone or in combination with 3-MA (5 mM) for 72 hours. **D** The expression levels of autophagy-lysosome-related proteins and apoptosis markers were analyzed by western blot in ACHN cells after SHMT2 knockdown following treatment with 3-MA (5 mM) for 72 hours. GAPDH served as loading control. **E** Cell proliferation assay of ACHN cells after SHMT2 knockdown following treatment with 3-MA (5 mM). Error bars represent SD (*n* = 3). **F** Heat-map of 20 differentially expressed cytochrome.*c*.oxidase subunits between shNC and shSHMT2 cells. Red represented significantly upregulated proteins and blue represented significantly downregulated proteins after SHMT2 knockdown. A represented shNC, B represented shSHMT2. **G** Western blot analysis of mitochondrial and cytosolic cytochrome *c* levels after SHMT2 knockdown in ACHN cells. GAPDH or HSP60 was used as the loading control for cytoplasm and mitochondria, respectively. *P < 0.05, **P < 0.01.

We next explored the effects of the overactivated autophagy-lysosome pathway on ccRCC cells. Autophagy serves as a dual regulatory mechanism directing cellular fate. In addition to its normal protective effects, numerous autophagosomes fuse with the lysosomes, resulting in leakage of lysosomal contents into the cytosol and increased LMP, which can trigger multiple cell death pathways, including apoptosis and necrosis [17, 18]. Recently, a galectin puncta assay was proved to be a sensitive method to detect LMP. Galectin is translocate from the cytosol to lysosomes upon LMP. Thus, we performed the galectin 1 puncta assay to confirm the occurrence of LMP after SHMT2 knockdown. As shown in Fig. 5A and Fig. S4A, control cells exhibited a weak and diffuse cytosolic fluorescence of galectin 1. In siSHMT2 cells, strong galectin 1 puncta and co-localization with LAMP1 were detected, suggesting lysosome-specific membrane damage. LMP also leads to a release of lysosomal proteases (cathepsins) into the cytosol. Immunofluorescence staining showed irregular cathepsin B-positive cytoplasmic aggregates in ACHN and A-498 cells treated with siSHMT2, which only partially colocalized with LAMP1 (Fig. 5B, S4B). In addition, western blot results also indicated that SHMT2 knockdown led to significant release of cathepsins into the cytosol (Fig. S5A). NEXT, we investigated the relationship between autophagy and LMP. The increased co-localization galectin 1 with LAMP1 in siSHMT2 cells was significantly abrogated after treatment with 3-MA (Fig. 5A, S4A). Similarly, 3-MA treatment also reduced the aggregates or diffusion of cathepsin B in siSHMT2 cells (Fig. 5B, S4B). In addition to pharmacological regulation, we also inhibited autophagy with Atg7 knockdown and found the similar results to the 3-MA treatment (Fig. S6A–C). Thus, these results suggested that excessive autophagy induced by SHMT2 knockdown in ACHN cells increased LMP, whereas inhibition of autophagy reduced LMP.

To further determine whether the autophagy and LMP caused by SHMT2 knockdown could induce apoptosis in ccRCC cells, flow cytometry analysis was conducted. SHMT2 knockdown significantly increased the apoptosis rate in ccRCC cells (Figs. 5C, S5B, S7A), and 3-MA treatment or Atg7 knockdown observably reduced apoptosis compared with the experimental group (Fig. 5C, S5B, S6D). Western blot also revealed the similar results: the protein levels of Bcl-2 (an antiapoptotic protein) were decreased and the levels of Bax (a proapoptotic protein) were increased after SHMT2 knockdown (Fig. 5D, S7B). While, after inhibition of autophagy, the protein expression profile was reversed (Fig. 5D, S6E). Similarly, 3-MA treatment obviously rescued the cell proliferation in siSHMT2 cells (Fig. 5E). These data suggest that SHMT2 knockdown promotes apoptosis in ccRCC cells maybe upon autophagy and LMP.

Based on the LC-MS/MS results, after SHMT2 knockdown in ACHN cells, 20 differentially expressed cytochrome. c. oxidase subunits were identified, among which 9 were markedly upregulated (Fig. 5F). Cytochrome c is a critical enzyme which can activate apoptosis. Once released from mitochondria into the cytoplasm, cytochrome c initiates the activation of caspase 3 and caspase 9. As expected, SHMT2 knockdown increased cytochrome c release (Fig. 5G). Moreover, cleavage of caspase 3 and 9 was also enhanced (Fig. 5E, S7B). Therefore, cytochrome c release may be another activator of apoptosis after SHMT2 knockdown.

### SHMT2 knockdown promoted metabolic reprogramming in ccRCC cells

**Fig. 6:**
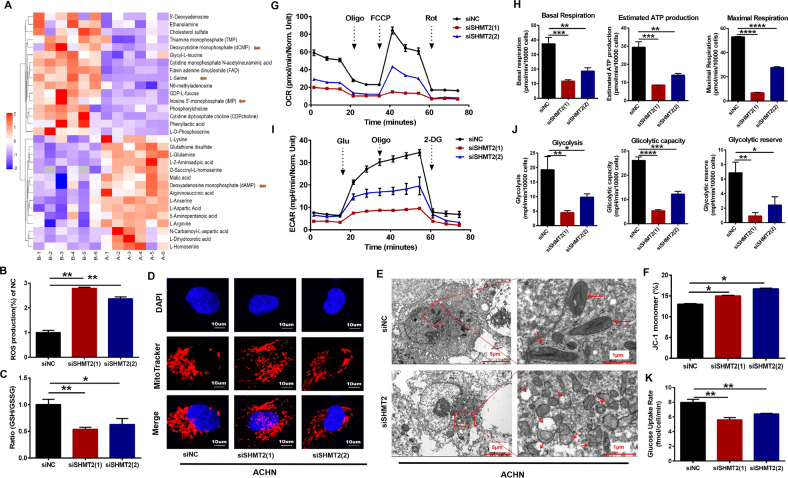
Effects of SHMT2 knockdown on metabolic reprogramming and mitochondrial structure in ACHN cells. **A** Heatmap of differentially accumulated metabolites between shNC and shSHMT2 ACHN cells based on the LC-MS metabolite profile. A represented shNC, B represented shSHMT2. **B** ROS labeled by DCF-DA were detected by flow cytometry after SHMT2 knockdown in ACHN cells. All values were normalized to siNC control values. **C** Cellular GSH/GSSG ratios were measured using a GSH/GSSG-Glo Assay kit. All values were normalized to siNC control values. **D** Representative confocal microscopy images of ACHN cells stained with Mito tracker (red) and DAPI nuclear staining (blue). Red fluorescence indicated the active mitochondria. **E** TEM analysis demonstrated the ultrastructure changes of mitochondria after SHMT2 knockdown in ACHN cells. Red arrows indicated mitochondria. The scale bars in the original image and the enlarged image represents 5 μm and 1 μm, respectively. **F** Quantification of the monomeric JC-1 levels, indicating a decrease in MMP after treatment with siSHMT2. **G** OCR measurements in ACHN cells after SHMT2 knockdown. **H** Basal respiration, estimated ATP production, and maximal respiration based on OCR results. **I** ECAR measurements in ACHN cells after SHMT2 knockdown. **J** Glycolysis, glycolytic capacity, and glycolytic reserve based on ECAR results. **K** Glucose uptake was measured using Promega glucose uptake-Glo assay kit.

Mitochondrial SHMT2 can catalyze the conversion of serine to glycine to fuel one-carbon pools, supporting tumorigenesis by the folate cycle, and the methionine cycle. We performed untargeted LC/MS analysis to detect different metabolite levels after SHMT2 knockdown in ACHN cells, especially nucleotides. A total of 114 metabolites were monitored, and the levels of 31 metabolites were changed significantly (Fig. 6A). After SHMT2 knockdown, blocking serine catabolism, serine and phosphoserine levels were markedly higher, and the contents of some nucleotides were also changed, including dCMP, IMP, and dAMP (Fig. 6A). Furthermore, the levels of FAD and glutathione disulfide (GSSG), which are both associated with one-carbon metabolism, were changed as well, suggesting overall metabolic disturbance.

One of the outputs of one-carbon metabolism is maintenance of the cellular redox balance. One-carbon metabolism was disrupted after SHMT2 knockdown in ACHN cells because serine catabolism was inhibited, and a stronger increase in ROS levels was observed than in control cells (Fig. 6B). This increase was associated with a decreased glutathione/glutathione disulfide (GSH/GSSG) ratio (Fig. 6C). MitoTracker staining showed that mitochondrial morphology changed from cord-like to dot-like (Fig. 6D). And TEM showed similar results: short swollen mitochondria and reduced mitochondrial ridges (Fig. 6E), indicating that the mitochondria were under stress and the mitochondrial structures were destroyed. Additionally, SHMT2 knockdown also led to a decrease in the mitochondrial membrane potential (MMP) as determined by flow cytometry (Fig. 6F), which is a landmark event in the early stage of cell apoptosis.

SHMT2 is a metabolic enzyme located in mitochondria. In order to examine whether SHMT2 knockdown affects mitochondrial metabolic function, Seahorse Mito Stress Tests were performed. The oxygen consumption rate (OCR) results showed that the basal and maximal respiration were significantly decreased in SHMT2 knockdown cells (Fig. 6G, H). In addition, the mitochondrial respiratory capacity and estimated ATP production were also reduced (Fig. 6G, H). As is well known, cancer cells prefer to use glycolysis to support cell growth, therefore, seahorse glycolysis stress tests were also performed to evaluate the glycolysis activity. The extracellular acidification rate (ECAR), which is associated with glycolysis, glycolytic capacity and glycolytic reserve, were also significantly decreased after SHMT2 knockdown (Fig. 6I, J). Moreover, there was a significant reduction in glucose uptake after SHMT2 knockdown. Collectively, these results suggest that SHMT2-mediated serine catabolism is important in maintaining global metabolic activity in ccRCC cells.

## Discussion

Metabolic reprogramming is an important hallmark of cancer [19]. Cancer cells sustain their survival and rapid proliferation by metabolic reprogramming, supplying not only a large amounts of energy, but also macromolecular substances. One-carbon metabolism supports the biosynthesis of nucleotides, proteins and lipids, important cofactors maintaining the cell redox balance, and SAM for methylation modification [7], which are crucial for cancer cell survival. The primary one carbon pool of one-carbon metabolism is the conversion of serine to glycine. The key metabolic enzymes are SHMTs, of which SHMT2 (mitochondria) is upregulated in a variety of tumors and closely related to tumor progression and patient survival. In the present study, we also revealed that SHMT2 was significantly upregulated in ccRCC tissues compared with controls and associated with patient survival. SHMT2 knockdown inhibited proliferation, migration, and invasion in ccRCC cells. While SHMT2 overexpression had the opposite effects, promoting tumor progression. SHMT2 depletion induced LMP via the autophagy-lysosome pathway, resulting in apoptosis, which affected the development of ccRCC. Furthermore, we explored the mechanisms underlying the suppressive effects of SHMT2 knockdown on ccRCC progression. Inhibition of SHMT2, a mitochondrial isozyme, destroyed mitochondria structure and function, disrupted one-carbon metabolism, and promoted metabolic reprogramming and oxidative stress, which increased LMP through excessive activation of the autophagy-lysosome pathway, leading to apoptosis.

Autophagy is an important metabolic pathway which maintains cell homeostasis in health and plays either a protective or a destructive role in disease [20, 21]. Although autophagy exhibits the prosurvival function under normal circumstances, when massively upregulated, autophagy has been demonstrated to promote cellular death in a wide variety of cancer [22–24]. In the present study, the lysosome pathway was the most significantly affected, and autophagic flux and the autophagy-lysosome pathway were also enhanced in siSHMT2 cells (Fig. 4), Thus, autophagy was massively upregulated in our study. Moreover, SHMT2 depletion inhibited ccRCC progression (Fig. 2 and S1). Therefore, autophagy played a destructive role in our results. While, under 5-FU treatment, SHMT2 deleption promotes autophagy and inhibits apoptosis in CRC cells by interacting with cytosolic p53 instead of its typical metabolic function [25], which was different from our results. In addition to metabolic function, we wanted to explore the deeper molecular mechanisms involved. Autophagy is a lysosome-dependent degradation process. Cancer cells are sensitive to lysosomal dysfunction and promote lysosome-dependent cell death upon LMP, which leads to apoptosis or necrosis [26, 27]. The lysosomal galectin puncta assay is a sensitive method to detect LMP [17]. A strong galectin 1 signal was detected in siSHMT2 cells (Fig. 5A), suggesting LMP, resulting in the release of lysosomal contents, such as cathepsins, into the cytoplasm (Fig. 5B, S5A). While, 3-MA treatment obviously reduced the LMP in siSHMT2 cells. One of the main downstream consequences of LMP is cathepsin-dependent Bid cleavage and promoting mitochondrial-dependent apoptosis [26, 28]. Bid activation leads to conformational alterations of proapoptotic Bcl2 protein and triggers a classic apoptosis pathway, including caspase activation, cytochrome c release, and MMP changes [29, 30]. In the present study, SHMT2 knockdown enhanced the cleavage of caspase 3 and caspase 9, and increased the cytochrome c release (Fig. 5D, F, G), which led to apoptosis (Fig. 5C). And inhibition of autophagy rescued the apoptosis and cell proliferation. Based on these results, we concluded that SHMT2 depletion overactivated autophagy, lots of autophagosomes fused with lysosomes, resulting in LMP, which induced apoptosis. While, How SHMT2 suppression leads to overactivation of autophagy remains to be elucidated?

SHMT2 knockdown could interrupt one-carbon metabolism, which suppresses carcinogenesis [31–34]. This phenomenon has not been reported in ccRCC. Only one study pointed out the clinical significance of SHMT2 in kidney cancer, but the underlying mechanism was not investigated [16]. In the present study, SHMT2 was certainly upregulated in ccRCC tissues (Fig. 1) and sustained ccRCC progression (Figs. 2 and 3). Untargeted metabolomics showed that the contents of some nucleotides were changed (Fig. 6A). Moreover, ROS levels were increased in siSHMT2 cells, which was associated with a decreased GSH/GSSG ratio, suggesting one-carbon metabolism and the redox balance were disturbed. A similar phenomenon was reported in previous studies [35, 36]. Interestingly, SHMT2 depletion destroyed the mitochondrial structure (Fig. 6D–F) and led to defective oxidative phosphorylation (OXPHOS) (Fig. 6G, H). A previous study showed that SHMT2 knockout resulted in a lack of the taurinomethyluridine base at tRNAs, causesing defective translation, which impaired expression of respiratory chain enzymes [37]. Based on genetic screens, SHMT2 null cells could not maintain formylmethionyl-tRNA pools and mitochondrial proteins, which were required for cell profiferation [38]. In addition, system-wide analysis demonstrated that the oxidation of one-carbon units derived from serine catabolism was a novel pathway for ATP synthesis via OXPHOS [39, 40]. These works revealed the mechanism linking serine catabolism to the OXPHOS modulation is complex. Here, consistent with the previous reports, OXPHOS activity was also decreased after deletion of SHMT2 in our work, which might be due to destruction of the mitochondrial structure and LMP, resulting in mitochondrial-dependent apoptosis. Cancer cells largely relied on anaerobic glycolysis as the main energy source to maintain their uncontrolled proliferation [5]. The enhancement of glycolysis promoted cell progression in various cancer types [41–43]. Well, we observed reduced glycolysis activity after SHMT2 knockdown in our results (Fig. 6I, J), which not only affected the yield of ATP, but also reduced the formation of glycolytic intermediates, affecting the biosynthesis of cellular components, such as amino acids, nucleotides, and lipids. Why did disruption of mitochondrial serine catabolism by SHMT2 deletion inhibit glycolytic flux? We showed that the ability of ccRCC cells to take up glucose decreased after SHMT2 knockdown, resulting in a decrease in glycolysis. Further research is needed to explore the specific underlying mechanism. Thus, based on the above results, we believe that the stress state caused by overall metabolic reprogramming after SHMT2 knockdown induced overactivation of autophagy in ccRCC.

In conclusion, we demonstrates that SHMT2 functiones as an oncogenic gene to promote ccRCC progression. After SHMT2 knockdown, cell homeostasis is disrupted because of the lack of ATP, disturbance of the biosynthesis of cellular components, and increased ROS levels, causing a state of extreme stress in ccRCC cells, eventually, autophagy is overactivated, which leads to apoptosis. Collectively, our work provides potential targets for the development of ccRCC treatment strategies.

## Materials and methods

### Cell culture and reagents

Unless otherwise stated, cell culture reagents were obtained from Gibco (Invitrogen) and chemicals were obtained from Sigma-Aldrich. The ccRCC cells were cultured in MEM (A498, ACHN), RPMI-1640 (786-O) or McCoy’s 5 A (Caki-2) supplemented with 10% FBS and 1% Pen/Strep. Cells were kept at 37 °C in humidified air with 5% CO_2_. The cells (except specially marked) were obtained from Procell Life Science &Technology Co.,Ltd (Wuhan, China).

### Animal experiments

All animal experiments were approved by the Biomedical Research Ethics Committee of Henan University. All animal procedures were in accordance with the guide of NIH for the care and use of laboratory animals. All animals were maintained in controlled pathogen-free IVC cages, 22 ± 2 °C, a relative humidity of 60 ± 5%, a 12/12-h light/dark cycle, and free access to food and water. To test the effects of SHMT2 on ccRCC cell proliferation in vivo, 5 × 10^6^ cells with stable SHMT2 knockdown or overexpression were injected subcutaneously into 6-week-old male nude mice randomly (*n* = 6/group, Charles River, Beijing, China). The tumor volumes were measured using digital calipers every 3 days and calculated using the following equation: tumor volume = 1/2 × length (mm) × width (mm)^2^.

### Clinical tissue specimens

Twelve pairs of ccRCC specimens and normal adjacent tissues were used to analyze SHMT2 RNA and protein levels. All patients were histopathologically diagnosed at Huaihe Hospital of Henan University. Before surgery, none of the patients received any chemotherapy or radiotherapy. All samples were frozen in liquid nitrogen immediately after surgical resection and stored at −80 °C until further use. Written informed consent was obtained from all patients prior to sample collection and the protocol was approved by the Biomedical Research Ethics Committee of Henan University.

IHC analysis of SHMT2 was performed on a TMA including 150 specimens from 75 ccRCC patients, which was obtained from OUTDO Biotech CO., Ltd. (Shanghai, China). After staining with anti-SHMT2 (33443, CST, USA), the staining intensity was recorded as 0–3, and the staining-positive rate was recorded as 0–4. The staining intensity and the staining-positive rate scores were multiplied, and the results were classified as follows: low (0–4), middle (5–8), and high (9–12).

### siRNA transfection and lentivirus infection

The siRNAs targeting human SHMT2 and nontargeting siRNA control were obtained from GenePharma (Shanghai, China). The siRNA sequences were as follows: siSHMT2 (1): 5′-GGAGAGUUGUGGACUUUAUTT-3′. siSHMT2 (2):5′-CUGGCCUCAUUGACUACAATT-3′. siNC: 5′-GGAGAGUUGUGGACUUUAUTT-3′. The siRNA targeting human Atg7 were obtained from Ribobio (5′-GGAGTCACAGCTCTTCCTT-3′, Guangzhou, China). The siRNAs were transiently transfected using lipofectamine RNAiMAX transfection reagent (Invitrogen, USA) according to the manufacturer’s instructions.

The lentivirus packaging system for stable knockdown or overexpression of SHMT2 was purchased from GenePharma (Shanghai, China). The stable cell lines were selected with puromycin (2 μg/ml) after lentivirus infection. The used shRNA target sequences were as follows: shSHMT2: 5′-GGAGAGTTGTGGACTTTAT-3′; shNC: 5′-TTCTCCGAACGTGTCACGT-3′.

### Cell proliferation assay

To measure the effects of the target gene on cell proliferation, cells were seeded in 96-well plates on day 1 after indicated treatments. According to the manufacturer’s instructions, the cells were incubated with 10 μl CCK-8 reagent in the dark for 2–4 h, and absorbance was measured at 450 nm using a spectrophotometer (Beckman, USA).

### Colony formation assay

After indicated treatments, cells were seeded in 6-well plates at low density (1000 cells/well) and cultured for 14 days. Cell colonies were fixed in 4% paraformaldehyde for 15 min, and 1 ml 0.1% crystal violet was added (Sigma, USA) to each well for 30 min at room temperature for visualization.

### Cell migration and invasion assays

Cell migration and invasion assays were performed respectively using transwell chambers (Millipore, USA) or transwell chambers coated with Matrigel Basement Membrane Matrix (BD, USA). First, 2 × 10^4^ or 4 × 10^4^ cells were seeded into the upper chamber with serum-free medium, and serum-containing medium was placed in the lower chamber. After 24 h, the nonmigrating cells and noninvasive cells were discarded, and the cells on the lower membrane surface were stained with 0.1% crystal violet solution (Sigma, USA) and observed using a microscope.

### Wound healing assay

A total of 2 × 10^5^ ccRCC cells with stable knockdown or overexpression of SHMT2 were seeded into 6-well plates.The cells were allowed to grow to 100% confluency, and a scratch was made using a P200 pipette tip. Images were collected at 0, 12, and 24 h under an inverted microscope. Cell migration was analyzed using NIH ImageJ software.

### Protein preparation and western blot analysis

Clinical tissue specimens and cells were lysed in RIPA lysis buffer (Beyotime, Shanghai, China) and the protein concentration was measured using a BCA Protein Assay Kit. About 20 μg of protein per lane was separated by SDS-PAGE, and then proteins were transferred to a PVDF membrane. After blocking in 5% BSA for 1 h, the membranes were incubated overnight with the primary antibodies. Next day, the membranes were incubated with secondary antibodies (Proteintech, China) at room temperature for 2 h. The enhanced chemiluminescent (Thermo Fisher, USA) was used to visualize the target bands.

Antibodies against SHMT2 (11099-1-AP), LAMP1 (21997-1-AP), LAMP2 (66301-1-AP), Caspase-3 (19677-1-AP), Caspase-9(10380-1-AP), P62 (18420-1-AP), cytochrome C (66264-1-Ig), Atg7 (10088-2-AP) and GAPDH (60004-1-lg) were purchased from Proteintech (China). Antibodies against LC3 (12741), cathepsin B (31718), cathepsin D (69854), Bcl-2 (15071), Bax (41162), PARP-1 (9532), cleaved PARP-1 (5625), and HSP60 (12165) were purchased from CST (USA).

### Confocal immunocytochemistry and the mRFP-GFP-LC3 system

To detect the formation of autolysosome, the co-localization of LC3 (an autophagosome marker) and LAMP1 (a lysosomal membrane marker) was examined by immunofluorescence assays. Briefly, ccRCC cells were seeded in confocal dishes after treatment for 24 h. Then cells were fixed with 4% paraformaldehyde for 10 min, and permeabilized with 0.2% Triton X-100 in PBS for 10 min. Between each steps, cells were washed with PBS. After blocking in 5% BSA in PBS for 1 h, cells were double immunostained with antibodies against LC3 and LAMP1 overnight, followed by incubation with the corresponding secondary antibodies. DAPI was added to stain nuclei. Cells were imaged by laser confocal scanning microscopy.

To measure LMP, antibodies against galectin-1 and cathepsin B/D/L antibodies were used for immunostaining. The immunocytochemistry protocol was as the above described.

To monitor autophagic flux, the mRFP-GFP-LC3 adenovirus was obtained from Hanbio Inc. (Shanghai, China). Briefly, cells infected by the adenovirus for 24 h, were subjected to different treatments and counterstained with DAPI. Fluorescence images were taken by laser confocal scanning microscopy. mRFP was used to label and track LC3. Weak GFP signals could indicate the fusion of autophagosomes and lysosomes to form autolysosomes, because GFP fluorescent protein was sensitive to acid. When autophagosome fused with lysosome, GFP fluorescence was inhibited, and only red fluorescence could be detected. The yellow spots (red/green merge) were autophagosomes, and red spots indicated autolysosomes.

### Flow cytometric analysis of apoptosis and the mitochondrial membrane potential (MMP)

Cells were collected and double stained with Annexin V and 7-AAD (BD, USA) for to quantify the apoptosis rate. Cells were stained with the fluorescent dye JC-1 (Beyotime, China) for analysis of the MMP. Flow cytometry analysis (Calibur, BD) was conducted following the manufacturer’s instructions. A strong monomeric JC-1 signal indicated a low MMP.

### Transmission electron microscopy

After treatment, cells were collected, fixed for 2 h with TEM fixative, washed in PBS (pH 7.4) for three times, and pre-embedded in 1% agarose solution. Then the cells were post-fixed with 1% osmium tetroxide in 0.1 M PBS (pH 7.4) for 2 h at room temperature. After dehydration in a gradient series of ethanol, the cells were embedded in pure EMBed 812 and cut into 60–80 nm thick ultrathin sections. After staining, the samples were examined by TEM (HITACHI).

### ROS measurement by flow cytometry

The DCFH-DA assay was performed according to the manufacturer’s instructions. ccRCC cells were cultured overnight in 6-well plates and then transfected with siRNA for 48 h. Cells were incubated with 10 μM DCFH-DA for 30 min, and then washed twice with PBS, and the labeled cells were harvested by trypsinization and resuspended in PBS. Intracellular ROS levels were measured by flow cytometry.

### GSH and GSSG measurement

Levels of reduced (GSH) and oxidized (GSSG) in cells were measured using the GSH/GSSG-Glo Assay kit (Promega, USA). Briefly, cells were plated on 96-well plates overnight and transfected with siRNA for 48 h. After culture medium was changed to Hank’s buffer, cells were lysed with either Total or Oxidized Glutathione Reagent on a plate shaker for 5 min. Then the lysate was transferred to a white 96-well plate. Subsequent luciferin generation and detection reagents were added according to the manufacturer’s instructions. Finally, luminescence was detected using a Varioskan Flash microplate reader (Thermo Fisher, USA).

### Seahorse extracellular flux analysis

Mitochondrial stress tests were conducted to measure the OCR of cells by sequentially adding drugs targeting the mitochondrial electron transport chain (ETC) to obtain key parameters reflecting mitochondrial function. Glucose is converted to pyruvate in the cytoplasm and further to lactate, releasing protons in the extracellular environment, causing acidification of the extracellular environment. The glycolysis stress test measures the ECAR of cells.

The ECAR and OCR tests were performed using a Seahorse XF HS Mini Analyzer (Seahorse Bioscience) according to the manufacturer’s instructions. Briefly, cells transfected with siRNA were seeded in XF8 cell culture microplates and incubated for 48 h prior to analysis. For the determination of the OCR, oligomycin, FCCP, and rotenone were respectively added. For the determination of the ECAR, glucose, oligomycin, and 2-deoxyglucose (DG) were added. All OCR and ECAR results were normalized to the protein contents in each well. All data were viewed and analyzed using Seahorse Wave software for XF analyzers. The basal respiration, maximal respiration, and estimated ATP production were calculated based on OCR data obtained from the mitochondrial stress tests, whereas glycolysis activity, glycolytic capacity, and glycolytic reserve were calculated based on ECAR data obtained from the glycolysis stress tests.

### Metabolomics analysis by HILIC LC-MS/MS technology

To extract metabolites from cells, 400 μl chilled methanol:acetonitrile (2:2, v/v) was added to 100 μl of each sample (1 × 10^7^cells). The mixture was vortexed for 60 s and then ultrasonicated for 30 min at 4 °C twice. The mixture was incubated at −20 °C for 1 h and then centrifuged at 14,000 rcf for 20 min at 4 °C. Finally, the liquid phase (supernatant) of each sample was transferred into a new tube for HILIC LC-MS/MS analysis. Samples were analyzed using an AB5500 QqQ mass spectrometer (AB Sciex,USA) coupled with a Waters I-class HPLC system (Waters, Ireland). Data acquisition and analysis were performed by Shanghai Applied Protein Technology Co., Ltd. The protocol was previously described [44].

### Quantitative proteomic analysis by tandem mass tag (TMT) technology

Tandem mass tag-based quantitative proteomic analysis was performed by Shanghai Applied Protein Technology Co., Ltd. First, 1 × 10^7^ cells were put into SDT lysis buffer (4% w/v SDS, 100 mM Tris/HCl, pH7.6, 0.1 M DTT) and the protein fraction was extracted. The protein levels in the supernatant was quantified with a BCA protein assay kit. An appropriate amount of protein was taken from each sample and trypsin digestion was performed by filter-aided proteome preparation (FASP), and then the peptide levels were quantified (OD280). Next, 100 µg peptide from each sample was labeled using TMT Isobaric Mass Tagging Kits (Thermo Scientific). The labeled peptides in each sample were mixed in equal amounts and graded using a High pH Reversed Phase Peptide Fractionation kit according to the manufacturer’s protocol. LC-MS/MS was performed on a Q-Exactive mass spectrometer (Thermo Scientific, USA) that was coupled to an Easy-nLC 1000 instrument (Thermo Scientific, USA). The procedures were previously described [44]. Protein identification and quantitative analysis were performed by Mascot 2.2 and Proteome Discoverer 1.4.

### Statistical analysis

Data were analyzed with GraphPad Prism software version 6. The results were presented as the mean ± SD. Immunofluoresence was quantified using NIH Image J. Comparisons between two groups were evaluated using the two-tailed Student’s *t*-test to determine significant *p*-values. *P < 0.05, **P < 0.01, and ***P < 0.001 were considered statistically significant.

## Supplementary information

Supplementary materials

Original Data File

aj-checklist

Certificate of English Language Editing

## Data Availability

All datasets generated and analyzed during this study are available from the corresponding author on reasonable request.
