# Correction: Serine hydroxymethyltransferase 2 knockdown induces apoptosis in ccRCC by causing lysosomal membrane permeabilization via metabolic reprogramming

**DOI:** 10.1038/s41419-023-05713-3

**Published:** 2023-03-06

**Authors:** Zhangnan Liu, Mengzhen Fan, Junqing Hou, Sijing Pan, Yanxin Xu, Hailong Zhang, Chen Liu, Xiangjun Hao, Xia Li, Huijuan Wang

**Affiliations:** 1 Joint National Laboratory for Antibody Drug Engineering, The First Affiliated Hospital of Henan University, School of Medicine, Henan University, Kaifeng, China; 2 Biotherapy Center, The First Affiliated Hospital of Zhengzhou University, Zhengzhou, China; 3 Henan Province Prostate Disease Prevention and Diagnosis Engineering Research Center, Henan University, Kaifeng, China; 4 Institute of Translational Medicine, School of Medicine, Henan University, Kaifeng, China

**Keywords:** Cancer metabolism, Cancer metabolism

Correction to: *Cell Death and Disease* 10.1038/s41419-023-05677-4, published online 20 February 2023

The original version of this article contained an error in authorship. After the publication the authors noticed that there should be a change in the order of authors. The correct order should be: Zhangnan Liu1,2,3,†, Mengzhen Fan1,†, Junqing Hou3, Sijing Pan1, Yanxin Xu1, Hailong Zhang1, Chen Liu1, Xiangjun Hao1, Xia Li1,4, Huijuan Wang1,4*. Xia Li no longer serves as co-corresponding author. The original article has been corrected.

