# Supplementary material for: Serine hydroxymethyltransferase 2 knockdown induces apoptosis in ccRCC by causing lysosomal membrane permeabilization via metabolic reprogramming
Source: Cell Death Dis. 2023 Feb 20;14(2):144. doi: 10.1038/s41419-023-05677-4 (PMC9941282; doi:10.1038/s41419-023-05677-4)
Supplement: Supplementary file 1 — Supplementary materials [file 41419_2023_5677_MOESM1_ESM.docx]

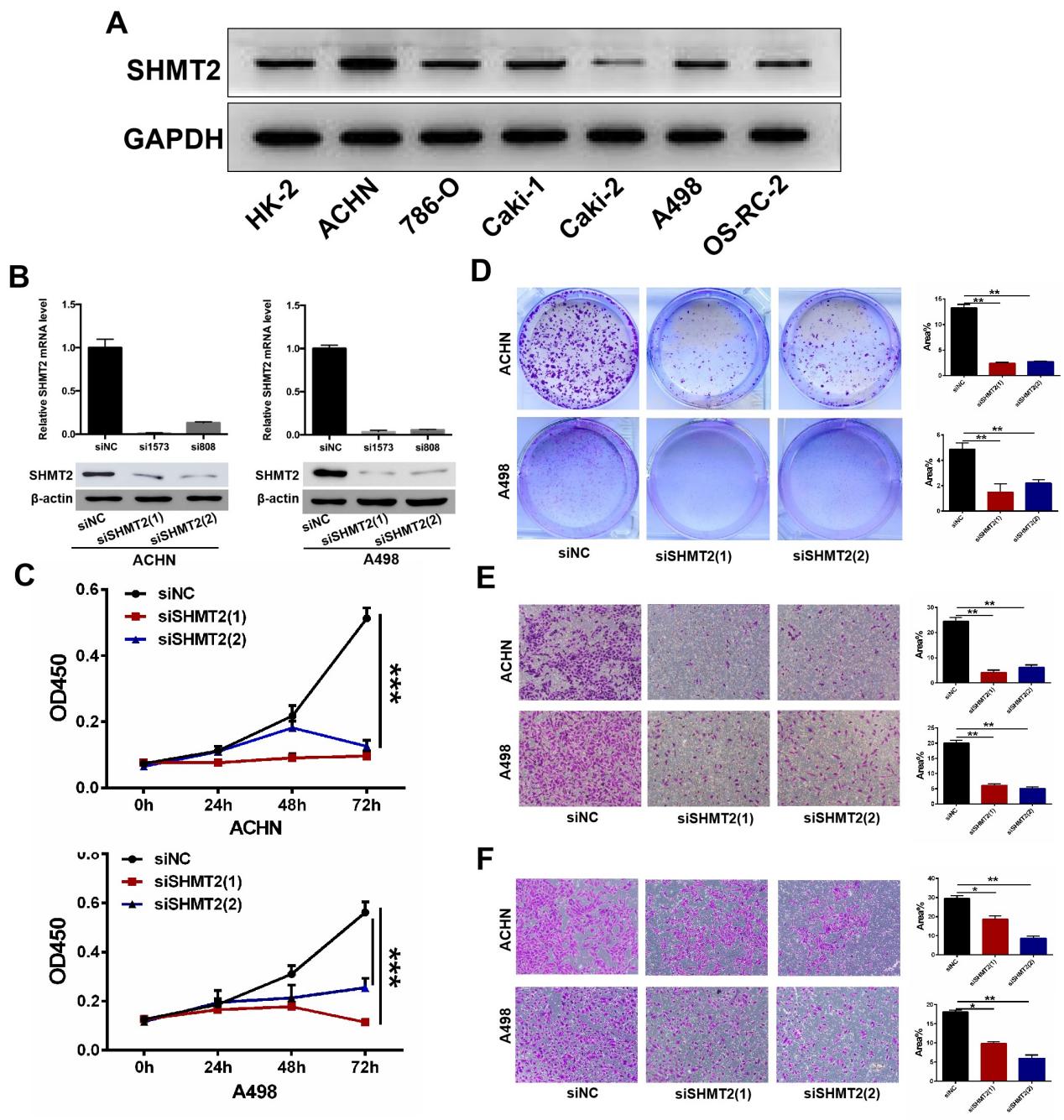


**Fig. S1 RNAi against SHMT2 inhibited the proliferation and metastasis of ccRCC cells in vitro.** ACHN and A498 cells were transfected with the indicated siRNAs. **A** Western blot analysis were performed to evaluate SHMT2 levels in multiple ccRCC cell lines. **B** After being transfected for 48 and 72 hours, qRT-PCR and western blot analysis were performed to evaluate SHMT2 level in ACHN and A498 cells. **C** Cell proliferation assay of ACHN and A498 cells transfected with siRNAs for 72 hours. Error bars represent SD (n=3). **D** Colony formation assays of ACHN and A498 cells transfected with siRNAs for 10 days. **E, F** Transwell migration (**E**) and matrigel invasion (**F**) assays of ACHN and A498 cells transfected with siRNA. *P < 0.05, **P < 0.01, ***P < 0.001


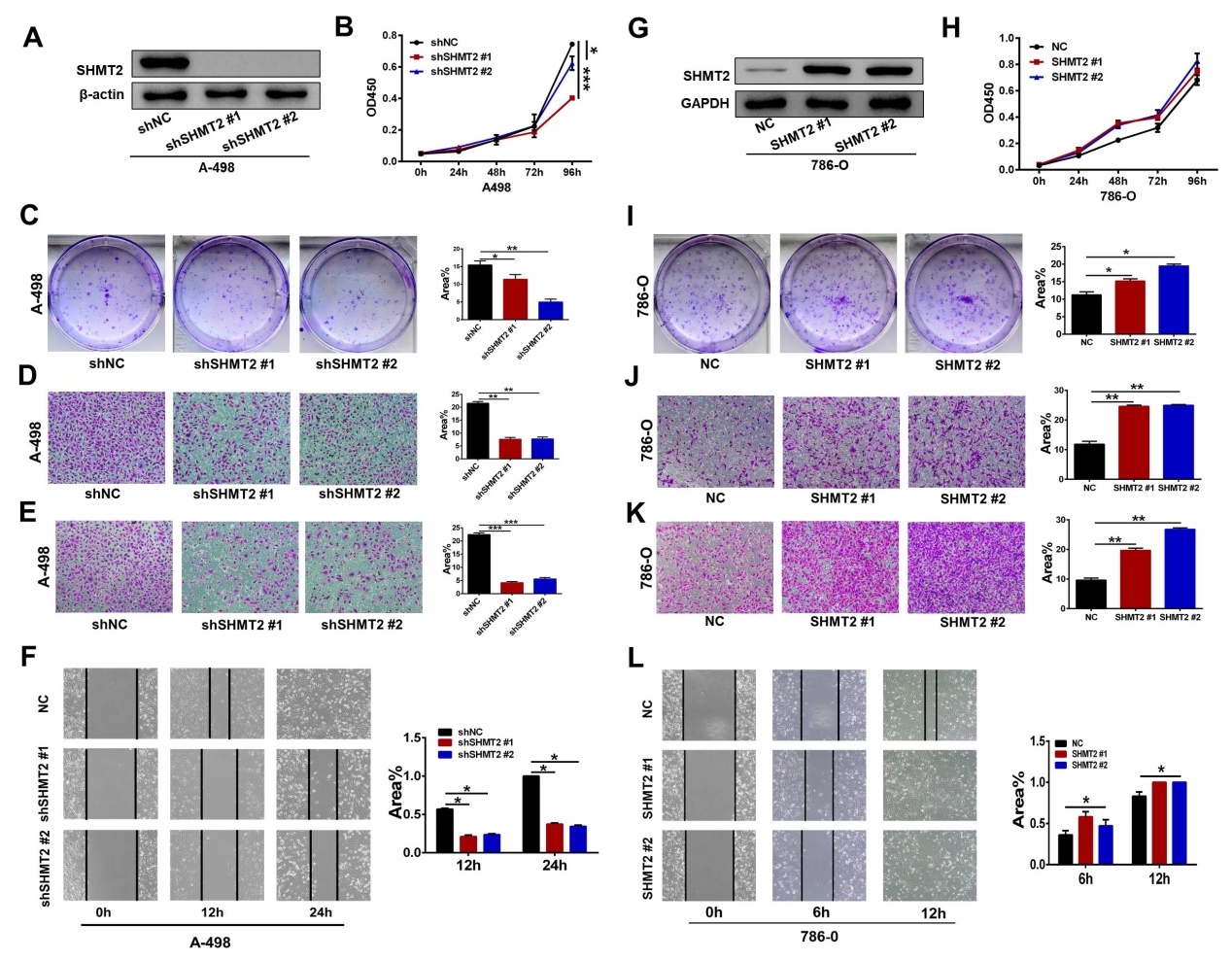


**Fig. S2 The effects of SHMT2 on proliferation and metastasis in ccRCC cells (A-498 and 786-O) in vitro.** **A-F** After stably being expressed by shSHMT2, the proliferation and metastasis of A-498 were inhibited in vitro. **G-L** After stably being overexpressed by SHMT2, the proliferation and metastasis abilities of 786-O were promoted in vitro. **A, G** The western blot was performed to evaluate SHMT2 protein level. **B, H** Cell proliferation assay of ccRCC cells. Error bars represent SD (n=3). **C, I** Colony formation assays of ccRCC cells for 10 days. **D, J** Cell migration assays of ccRCC cells through a 8 µm Transwel. e/k. Cell invasion assays of ccRCC cells through a Matrigel-coated 8 µm Transwell. **F, L** Wound-healing assay of ccRCC cells. *P<0.05,**P<0.01, ***P<0.001.


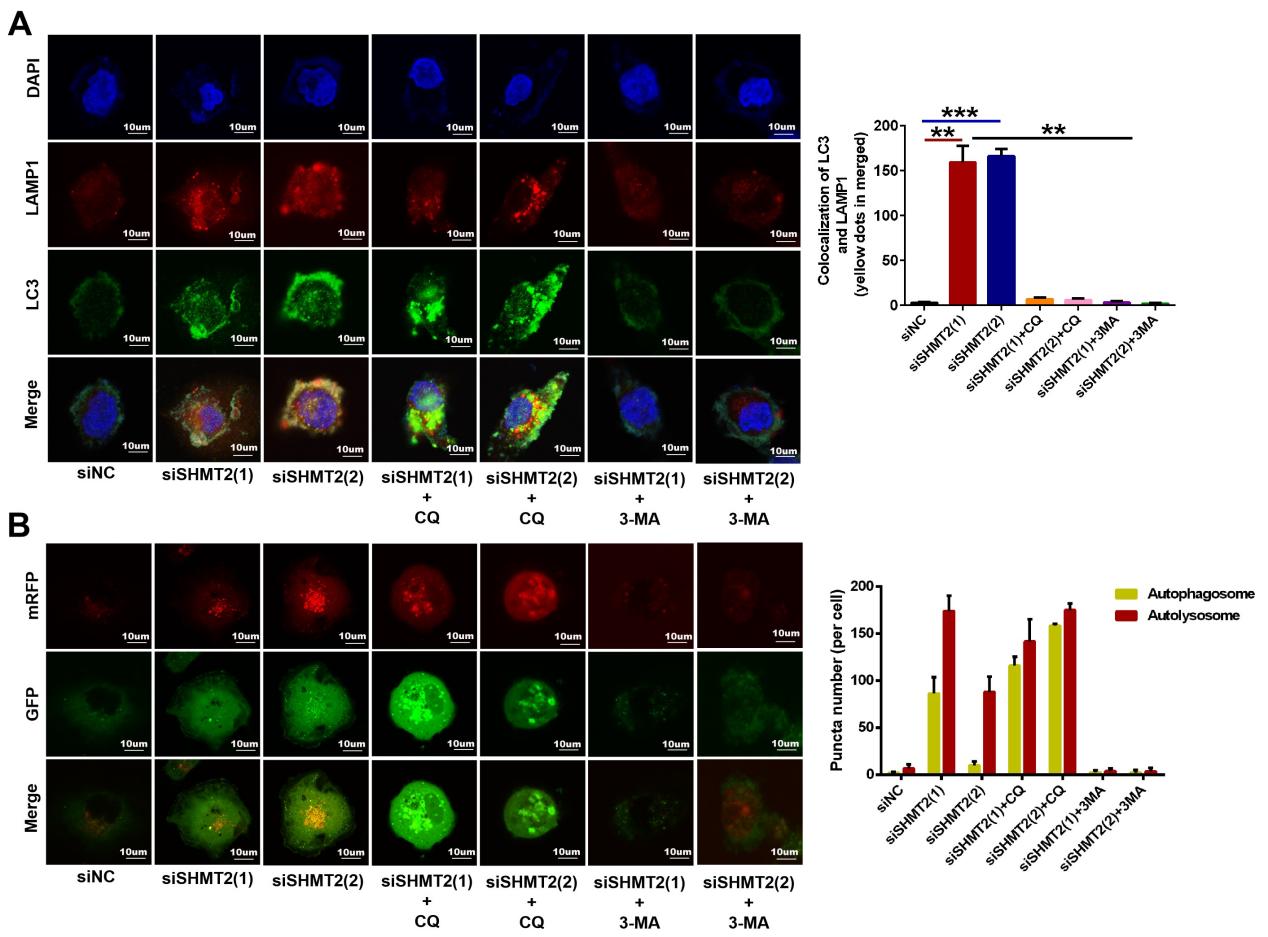


**Fig. S3 SHMT2 Knockdown activated the autophagy-lysosome pathway and affected autophagic flux in A-498 cells.** **A** Double immunofluorescence analysis of the co-localization of LC3 (green) and LAMP1 (red) in A-498 cells after SHMT2 knockdown following treatment with CQ or 3-MA. Nuclei were stained with DAPI(left). Quantification of colocalization of LC3 and LAMP1 (right). **B** A-498 cells were transfected with adenovirus harboring mRFP-GFP-LC3 (green and red puncta indicate GFP and mRFP, respectively) to follow autophagic flux after SHMT2 knockdown following treatment with CQ or 3-MA. Nuclei were stained with DAPI(left). Quantitative analysis of autophagosomes (yellow dots) and autolysosomes (red dots) (right).


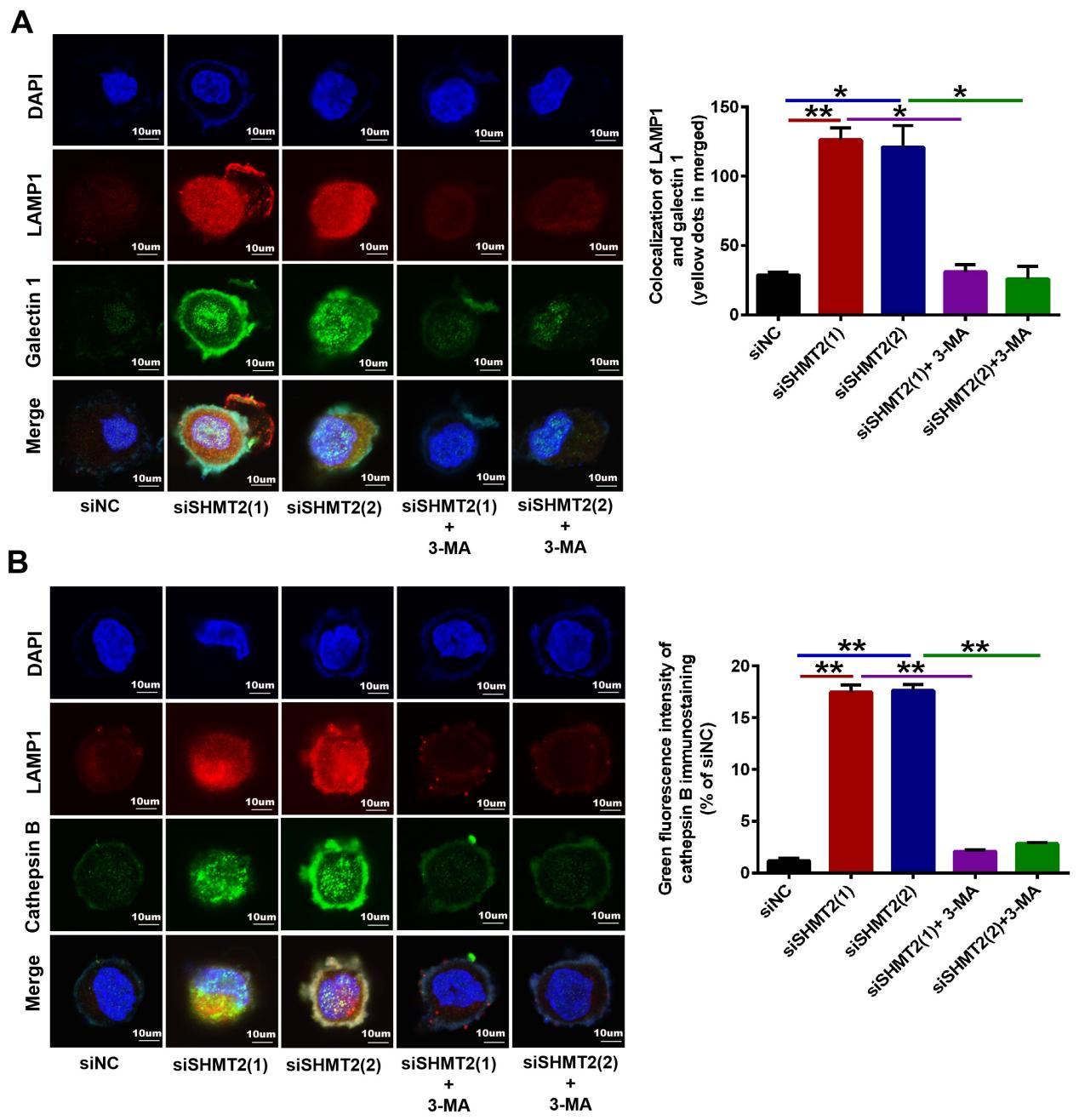


**Fig. S4 Inhibition of autophagy reduced SHMT2 Knockdown-induced LMP and apoptosis in A-498 cells.** **A** Double immunofluorescence analysis of the co-localization of galectin-1 (green) and LAMP1 (red) in A-498 cells after SHMT2 knockdown following treatment with 3-MA. Nuclei were stained with DAPI. Green Galectin punctea indicated compromised lysosomes (left). Co-localization of galectin 1 and LAMP1 was quantified using NIH Image J (right). **B** Double immunofluorescence staining of cathepsin B (green) and LAMP1 (red) in A-498 cells after SHMT2 knockdown following treatment with 3-MA. Nuclei were stained with DAPI (left). Quantification of green fluorescence intensity of cathepsin B immunostaining (right).


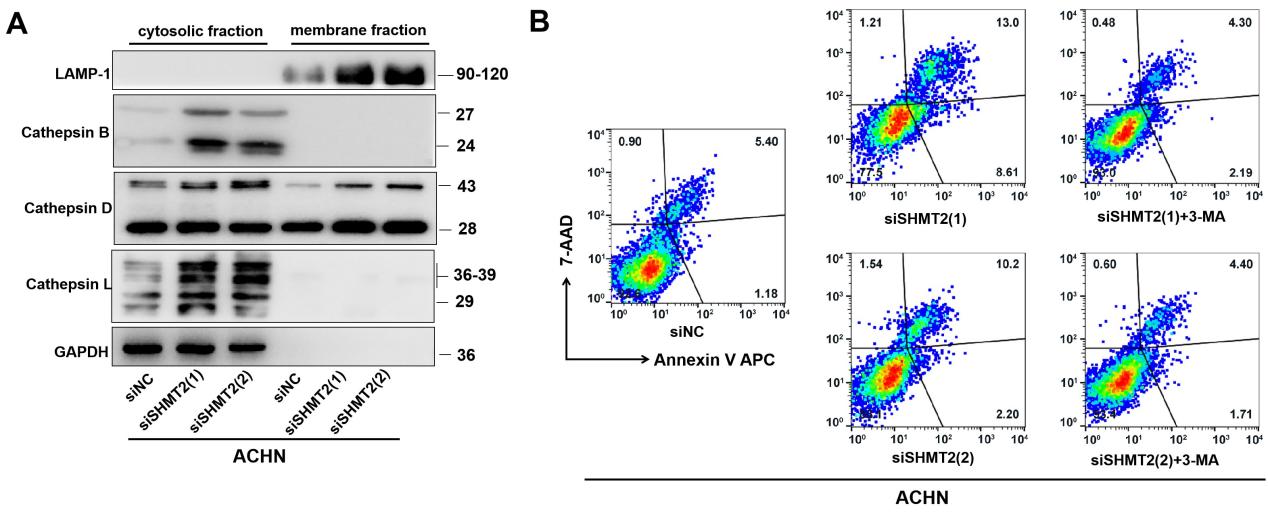


**Fig. S5 Knockdown SHMT2 induced LMP and cell apoptosis in ccRCC cells.** **A** The protein levels of Cathepsin B or D or L were invidated in different cell cytosolic and lysosomal fractions in ACHN cells. **B** Representative FACS results showed apoptosis after SHMT2 knockdown following treatment with 3-MA in ACHN cells.


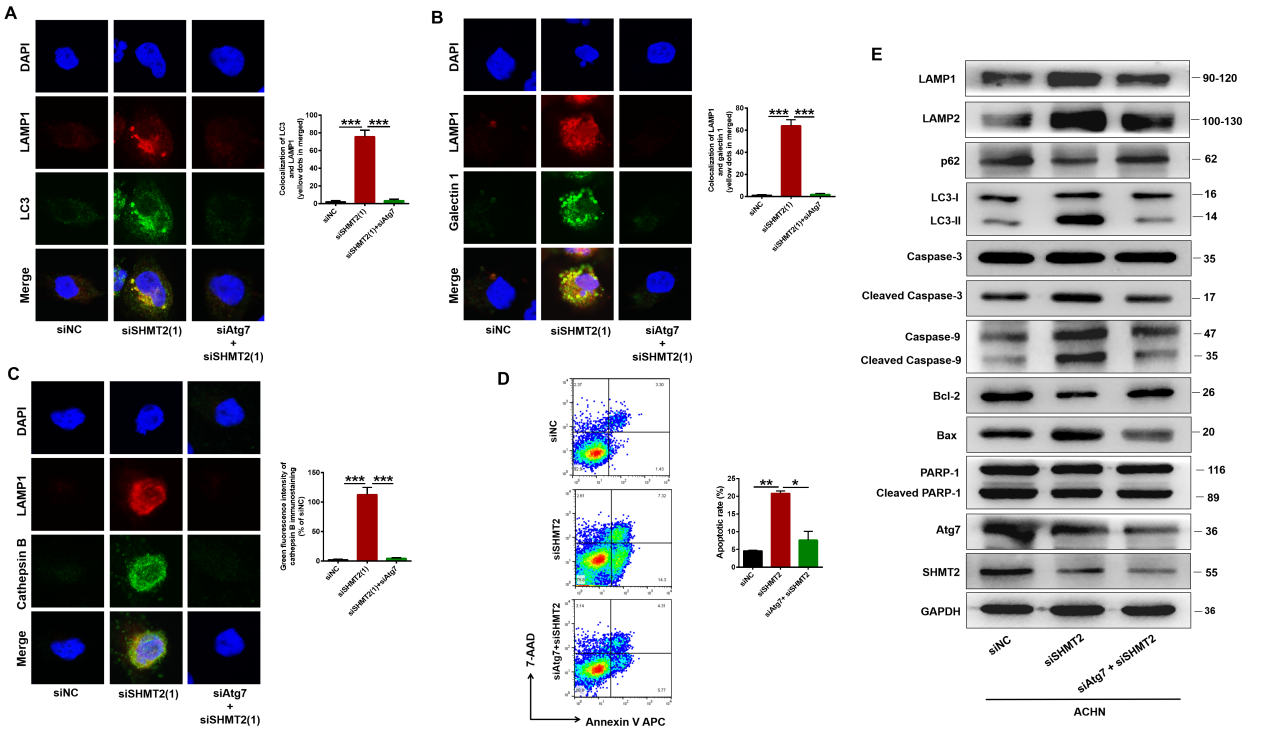


**Fig. S6 Inhibition of autophagy by Atg7 knockdown reduced SHMT2 Knockdown-induced LMP and apoptosis in ACHN cells.** **A** Double immunofluoresence analysis of the co-localization of LC3 (green) and LAMP1 (red) in ACHN cells after SHMT2 knockdown alone or in combination with Atg7 knockdown for 48 hours. Nuclei were stained with DAPI. **B** Double immunofluorescence analysis of the co-localization of galectin-1 (green) and LAMP1 (red) in ACHN cells after SHMT2 knockdown alone or in combination with Atg7 knockdown for 48 hours. Nuclei were stained with DAPI. Co-localization of galectin 1 and LAMP1 was quantified using NIH Image J (right). **C** Double immunofluorescence staining of cathepsin B (green) and LAMP1 (red) in ACHN cells after SHMT2 knockdown in combination with Atg7 knockdown for 48 hours. Nuclei were stained with DAPI. Quantification of green fluorescence intensity of cathepsin B immunostaining (right). **D** Representative FACS results showed apoptosis after SHMT2 knockdown in combination with Atg7 knockdown in ACHN cells (left). Quantitative FACS apoptosis results (right). **E** The expression levels of autophagy-lysosome-related proteins and apoptosis markers were analyzed by western blot in ACHN cells after SHMT2 knockdown in combination with Atg7 knockdown. GAPDH served as loading control.


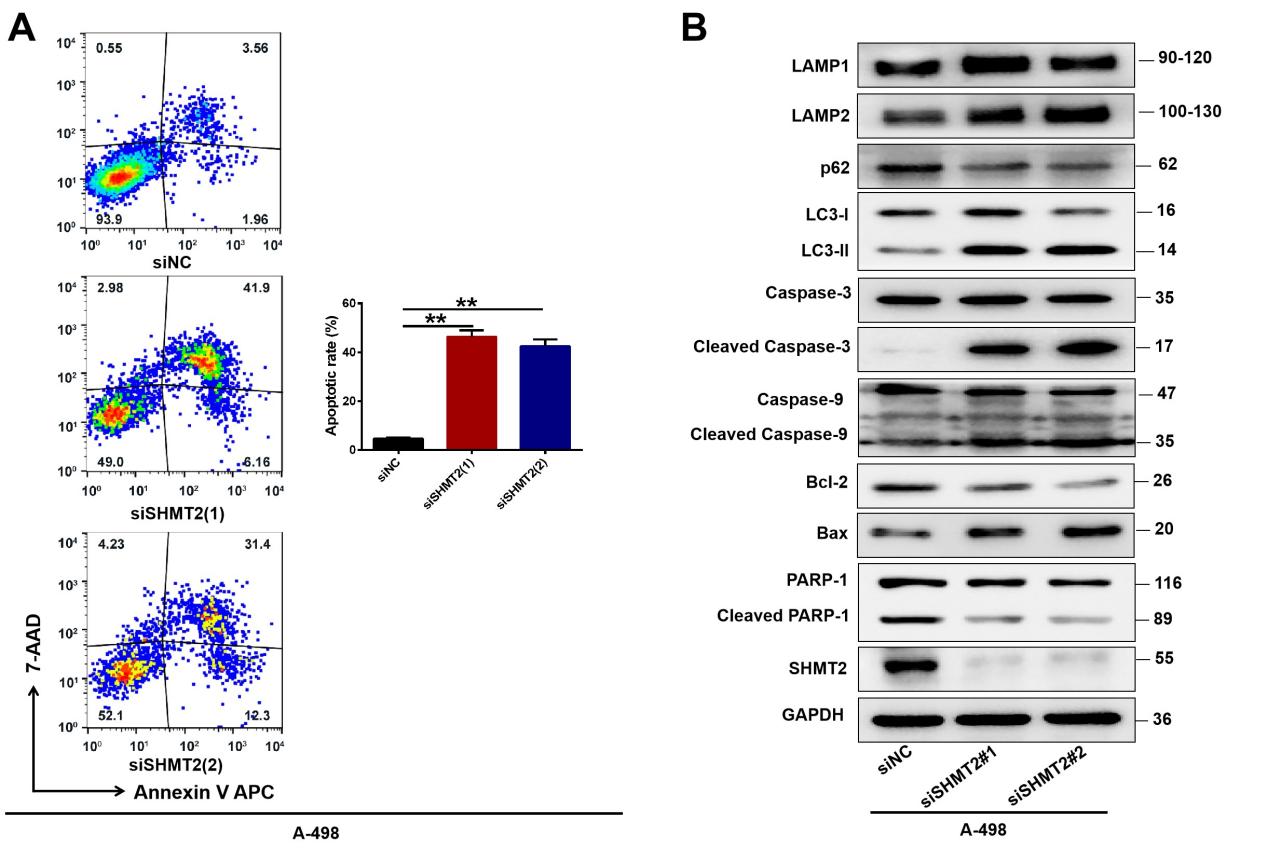


**Fig. S7 SHMT2 Knockdown induced apoptosis and autophagy in A-498 cells. A** Representative FACS results showing SHMT2 knockdown induced apoptosis in A-498 cells (left). Quantitative results showed that SHMT2 knockdown increased the percentage of apoptotic cells in A-498 cells (right). **B** The expression levels of autophagy-lysosome-related proteins and apoptosis markers were analyzed by western blot after SHMT2 knockdown in A-498 cells. GAPDH served as a loading control.
