# Supplementary material for: Serine hydroxymethyltransferase 2 knockdown induces apoptosis in ccRCC by causing lysosomal membrane permeabilization via metabolic reprogramming
Source: Cell Death Dis. 2023 Feb 20;14(2):144. doi: 10.1038/s41419-023-05677-4 (PMC9941282; doi:10.1038/s41419-023-05677-4)
Supplement: Supplementary file 2 — Original Data File [file 41419_2023_5677_MOESM2_ESM.pdf]

Figure 1C

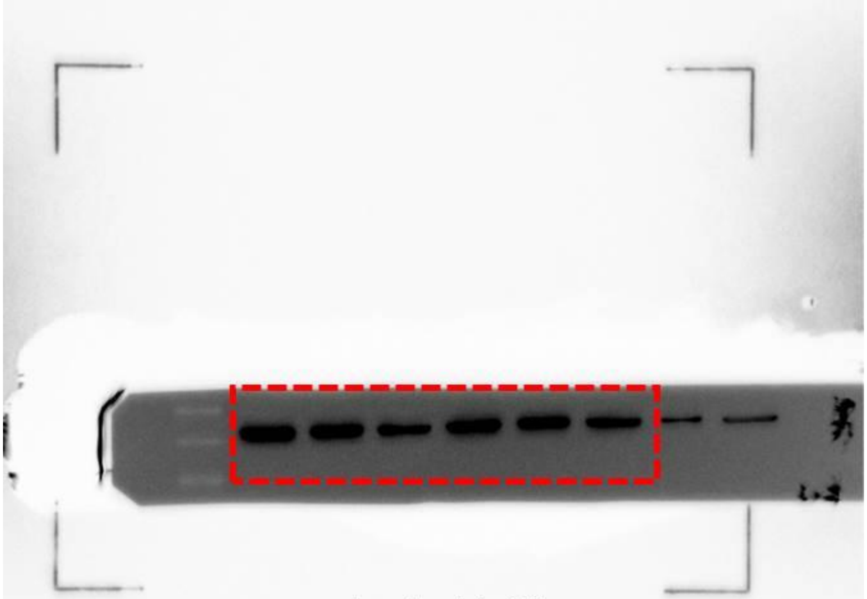

Actin (1-3)

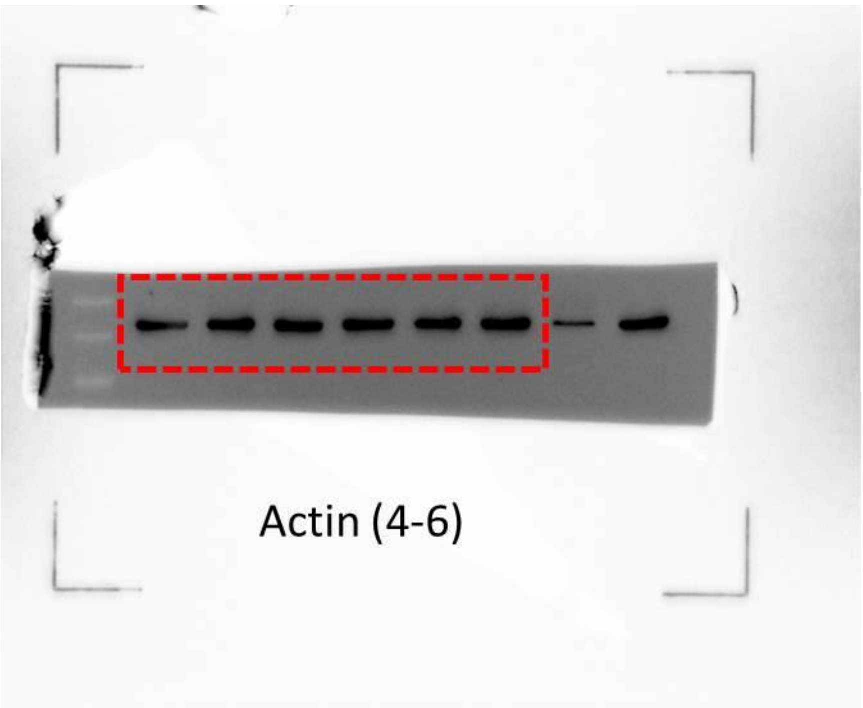

Actin (4-6)

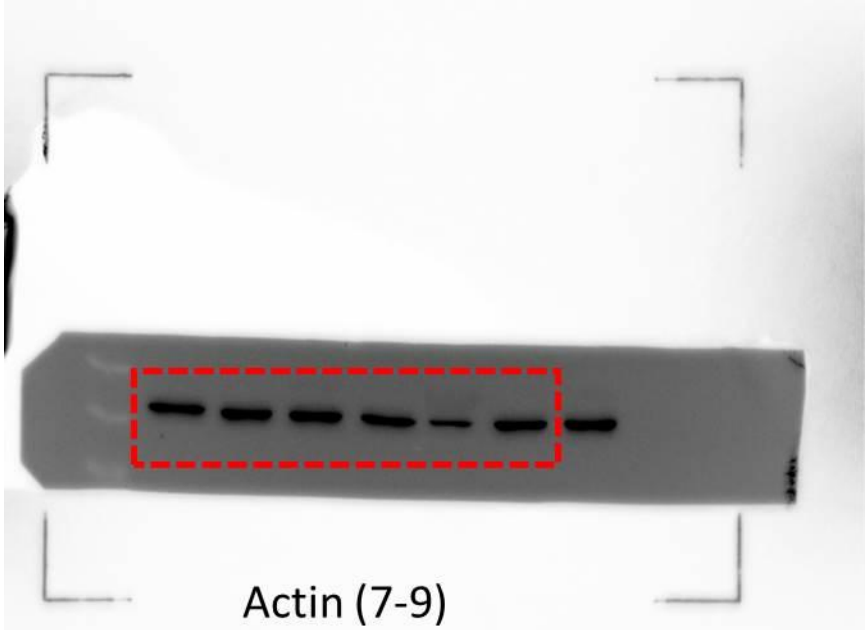

Actin (7-9)

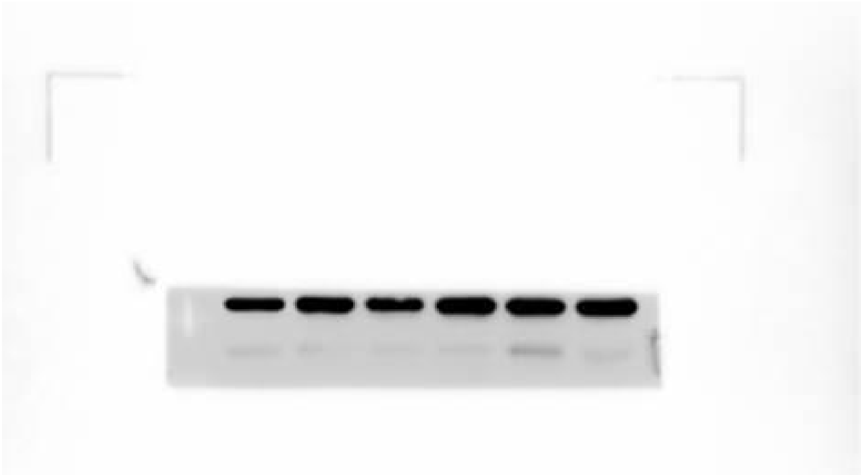

Actin (10-12)

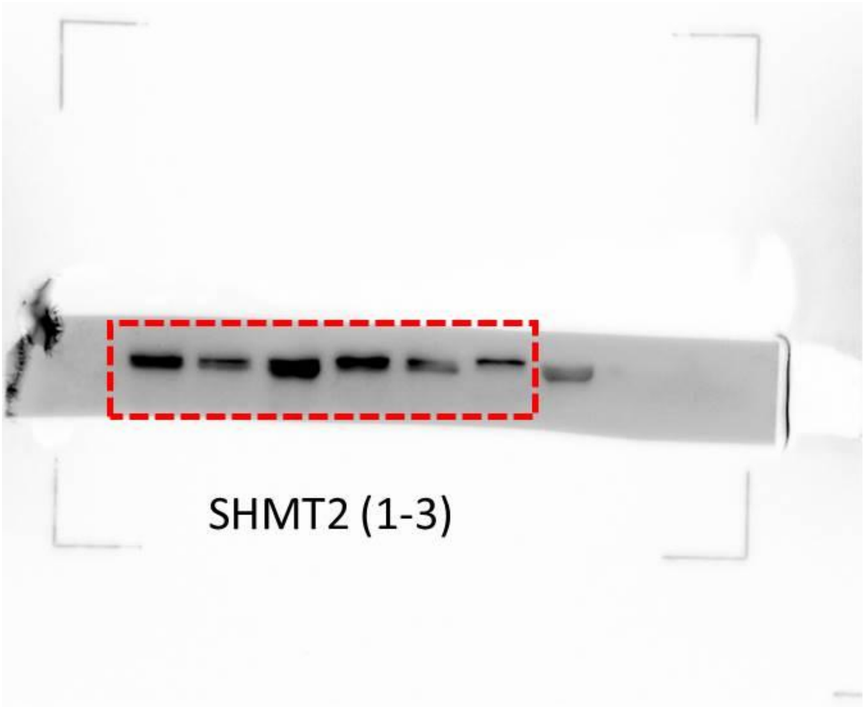
SHMT2 (1-3)

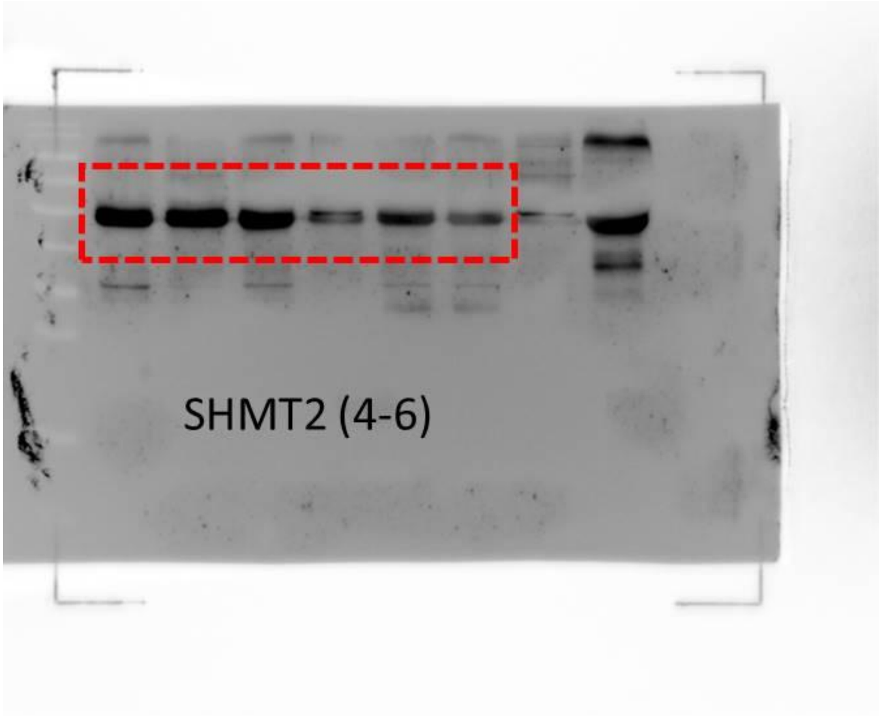
SHMT2 (4-6)

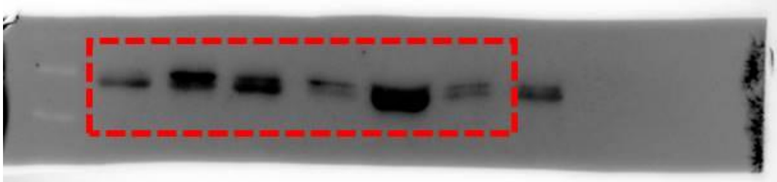
SHMT2 (7-9)

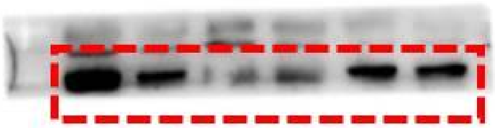
SHMT2 (10-12)

Figure 2A

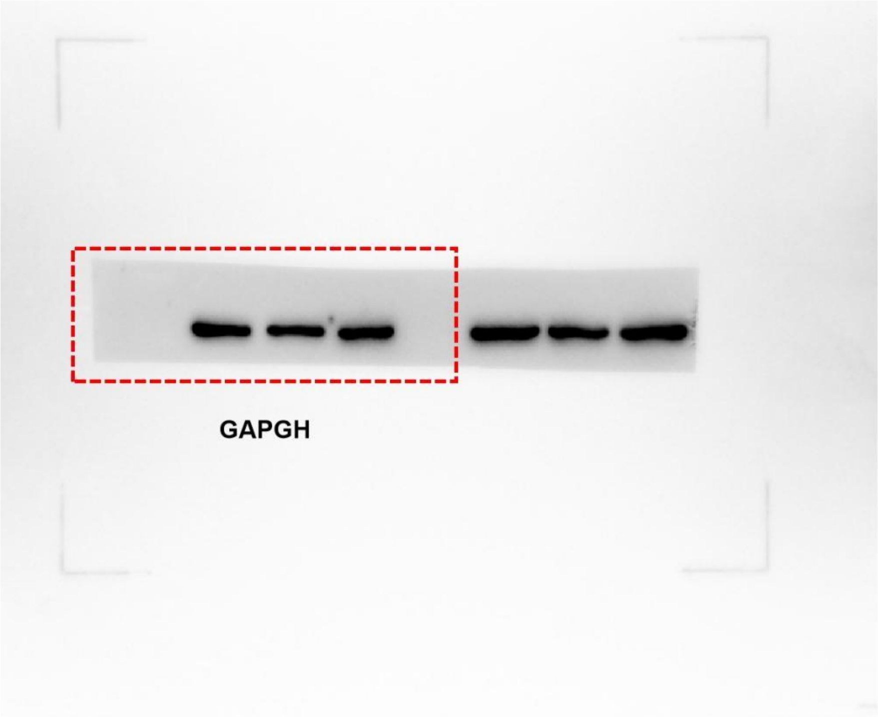

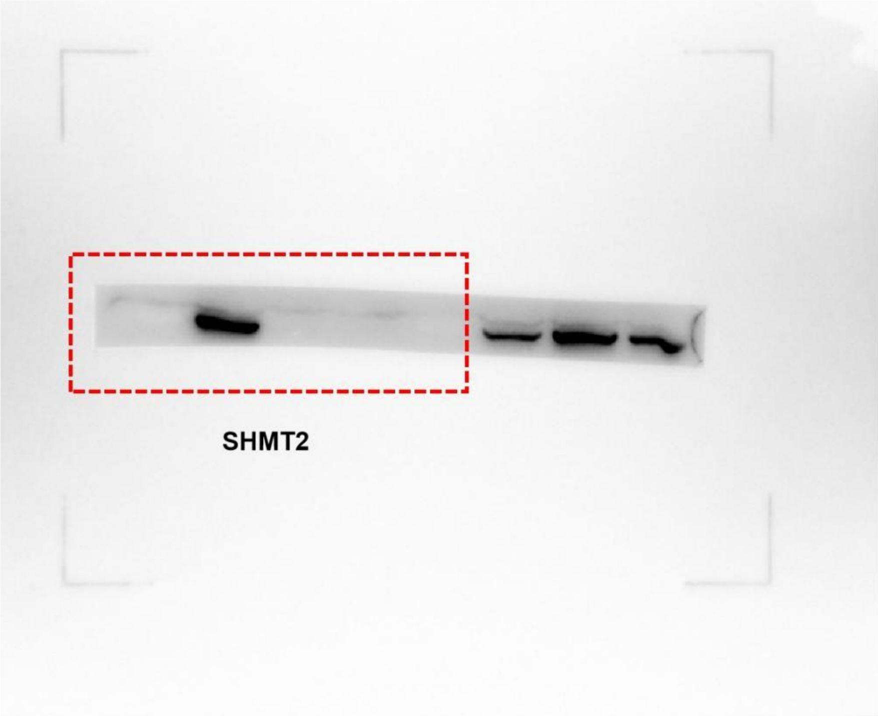

Figure 3A

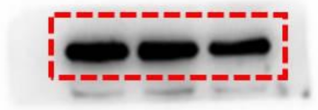

GAPDH

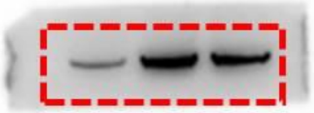

SHMT2

Figure 4C (ACHN-siSHMT2)

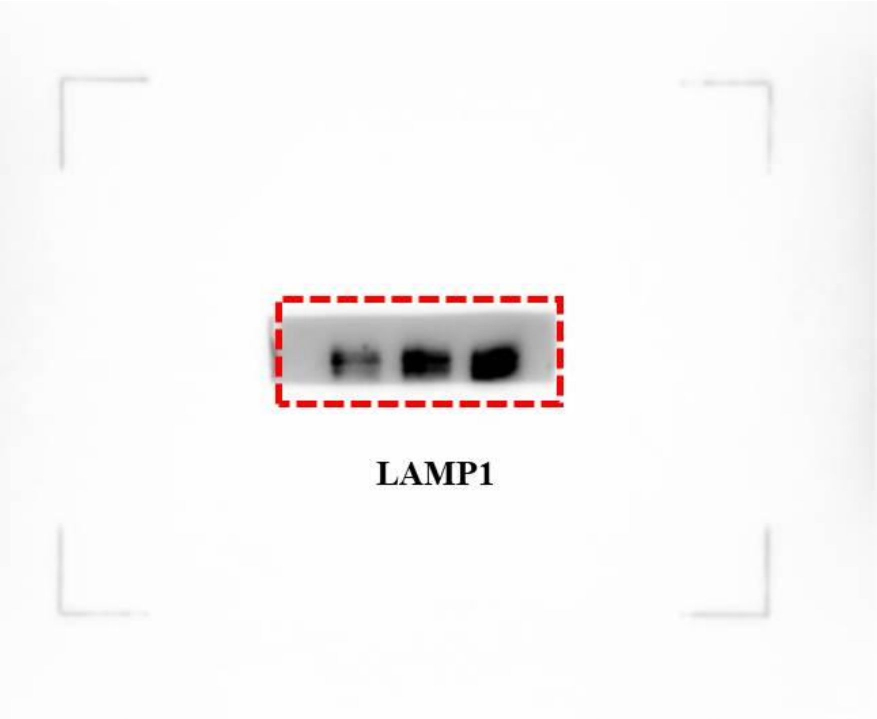

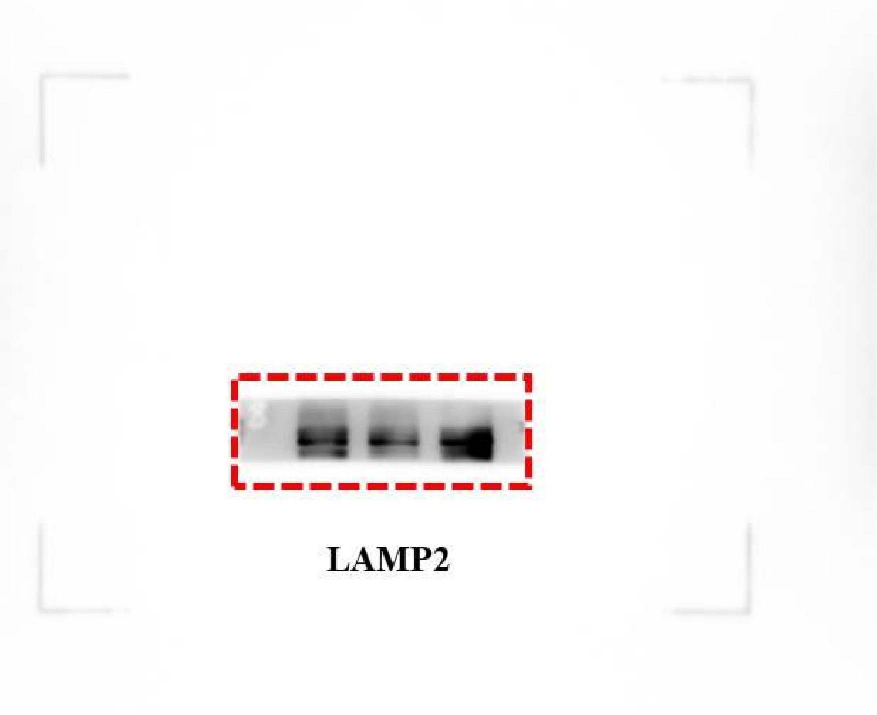

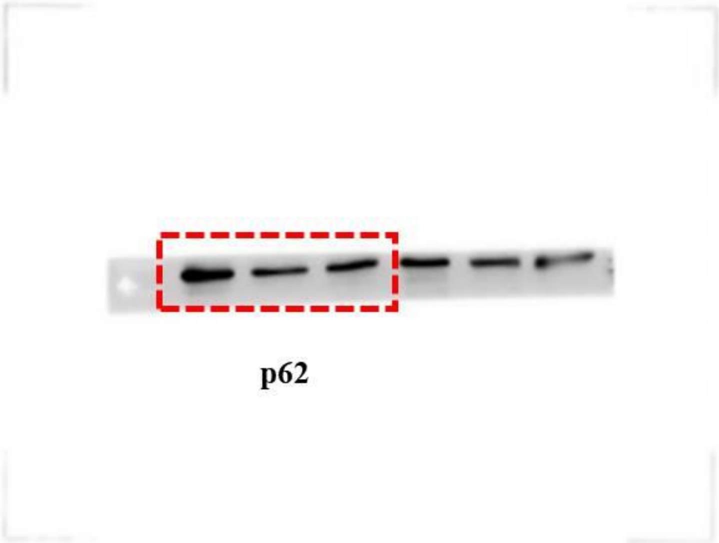

p62

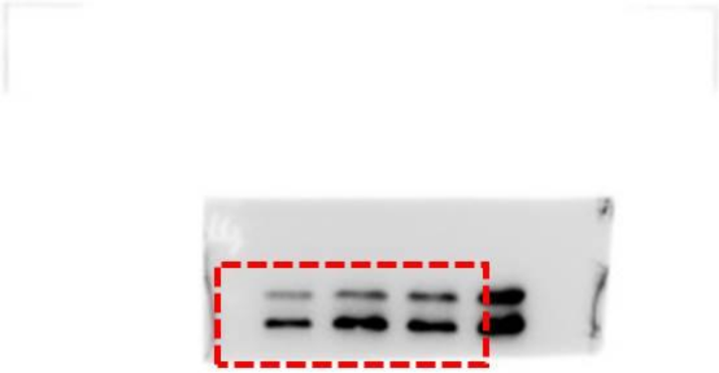

LC3

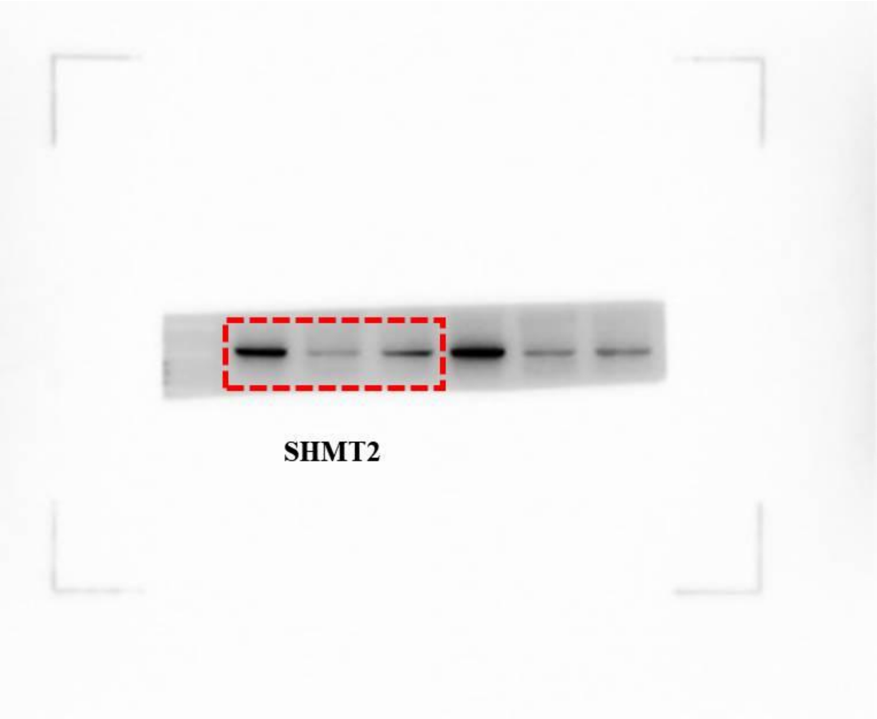

SHMT2

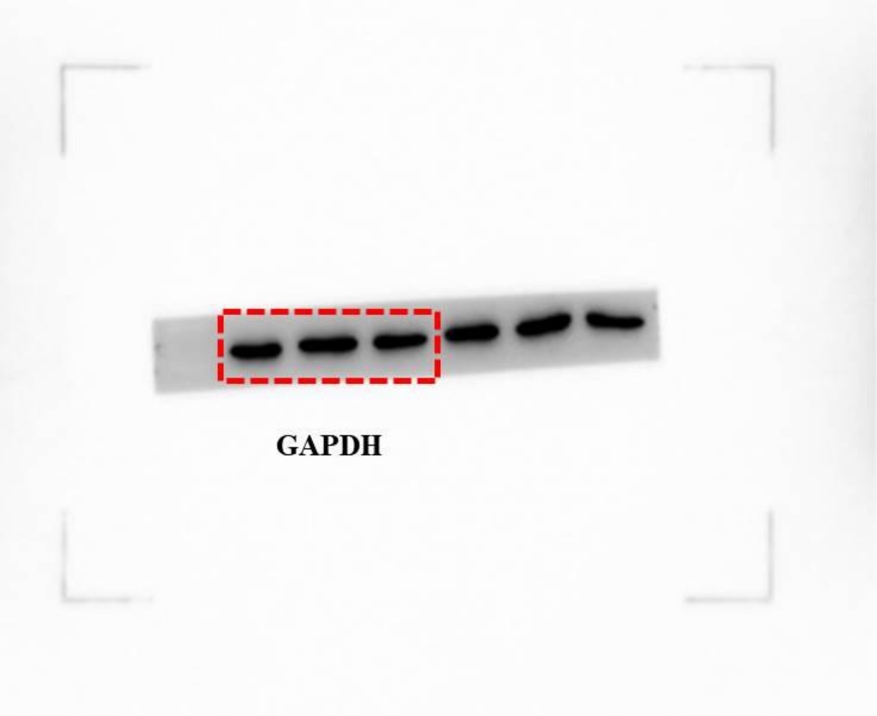

GAPDH

Figure 4C (ACHN-shSHMT2)

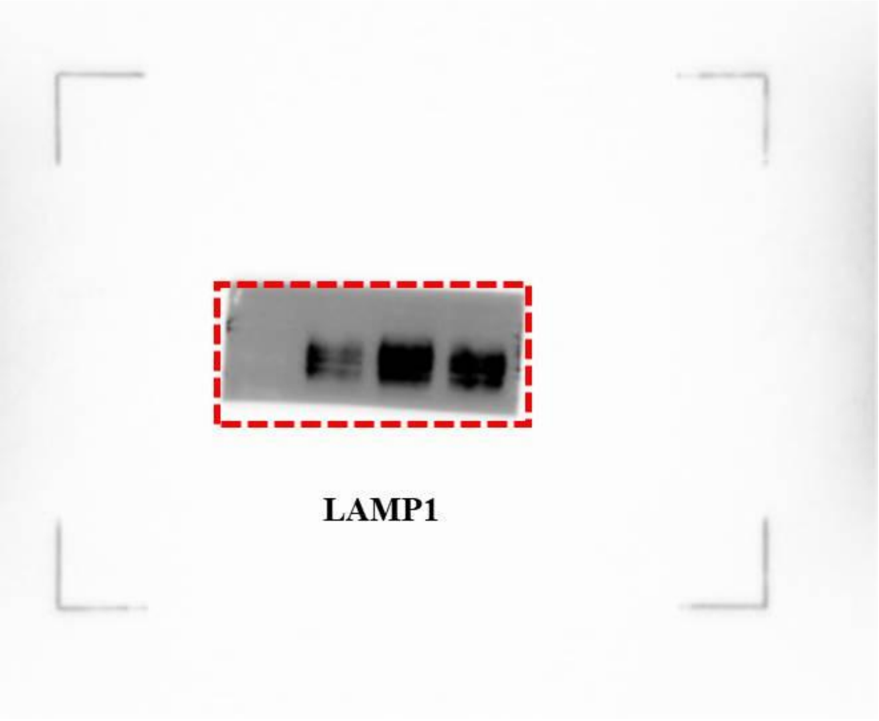

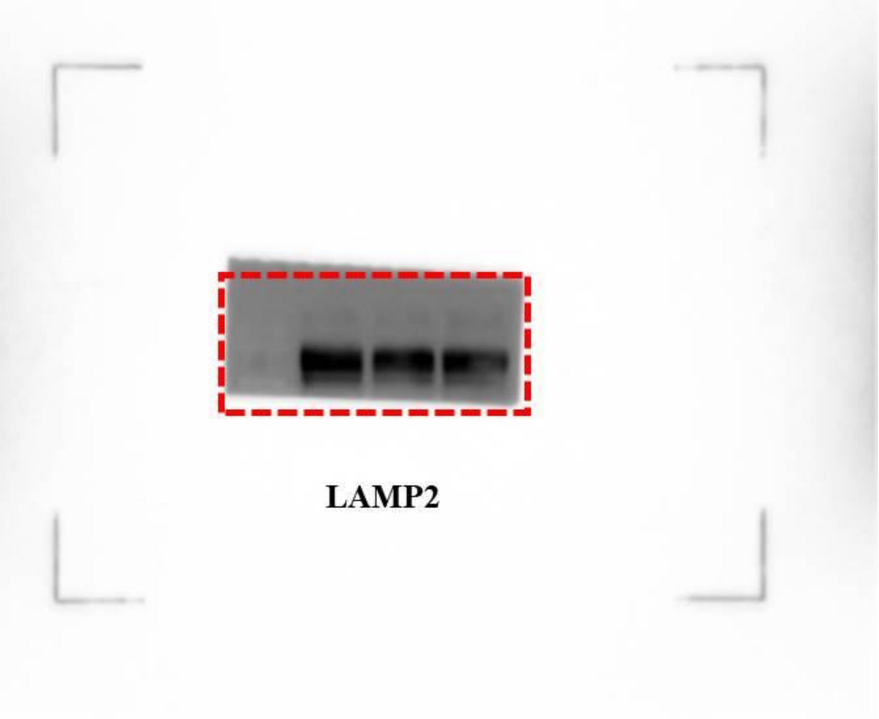

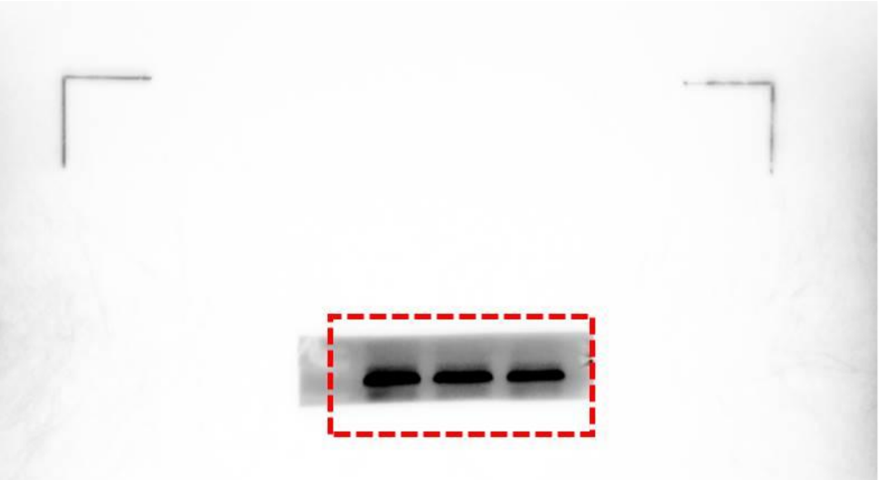

p62

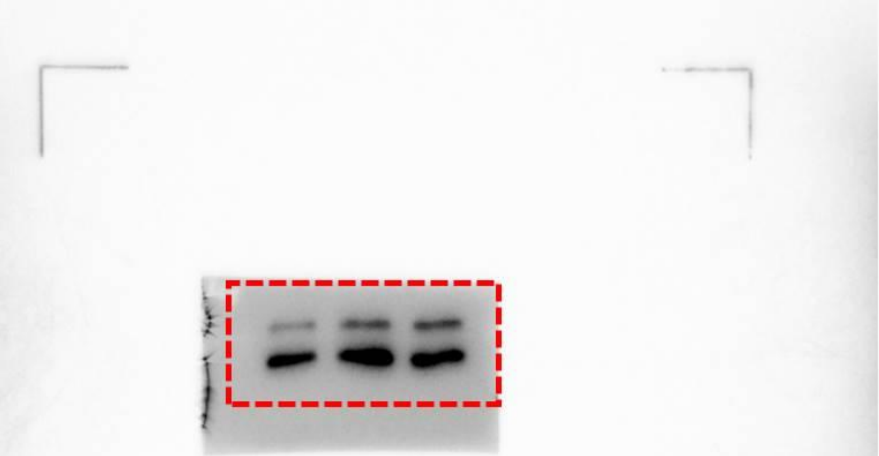

LC3

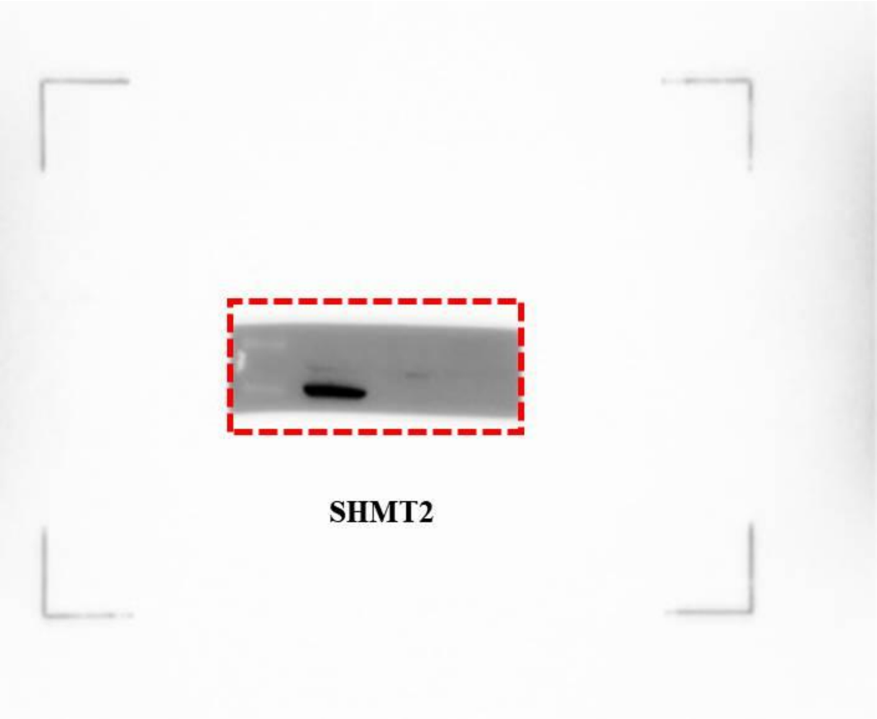

SHMT2

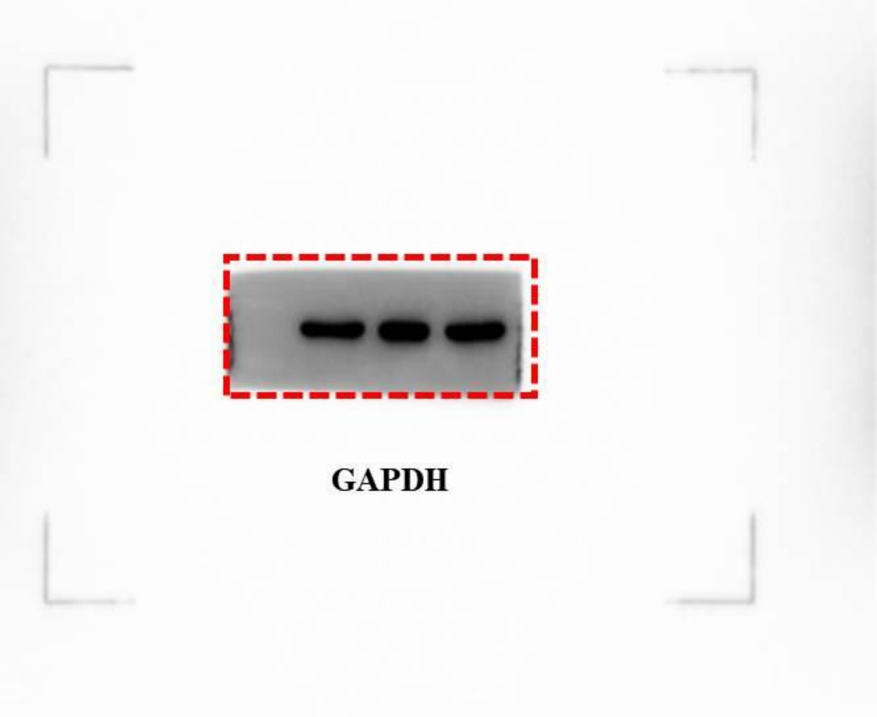

GAPDH

Figure 4C (Caki-SHMT2)

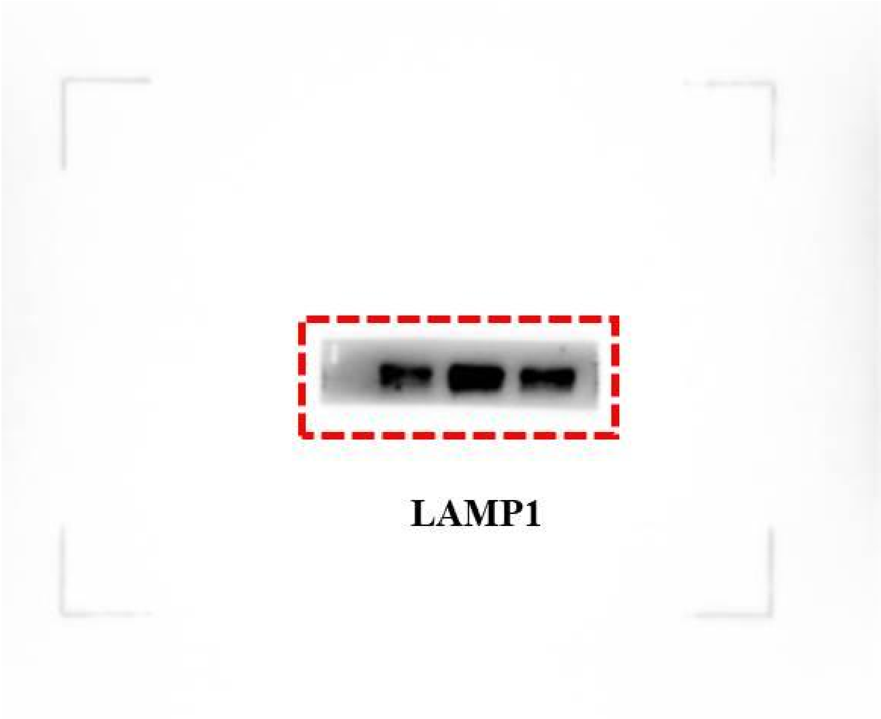

LAMP1

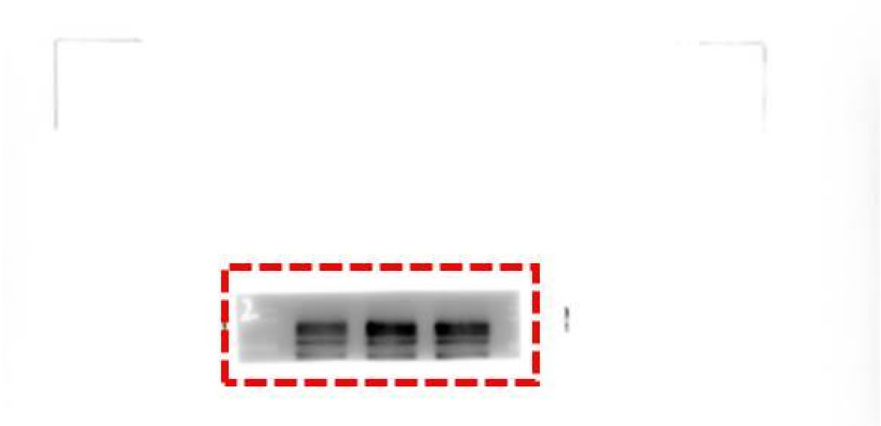

LAMP2

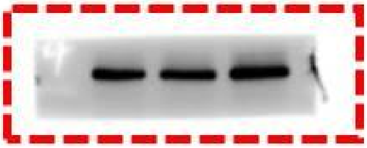

p62

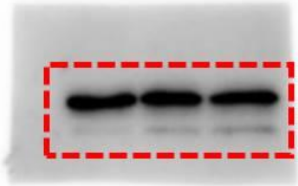

LC3

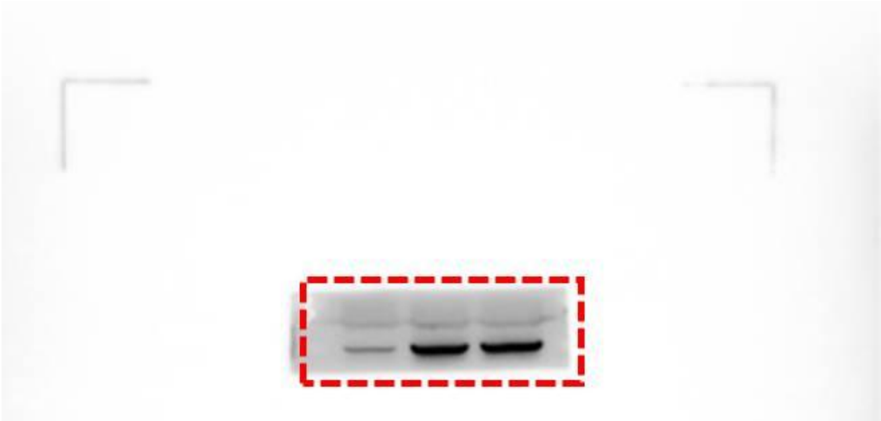

SHMT2

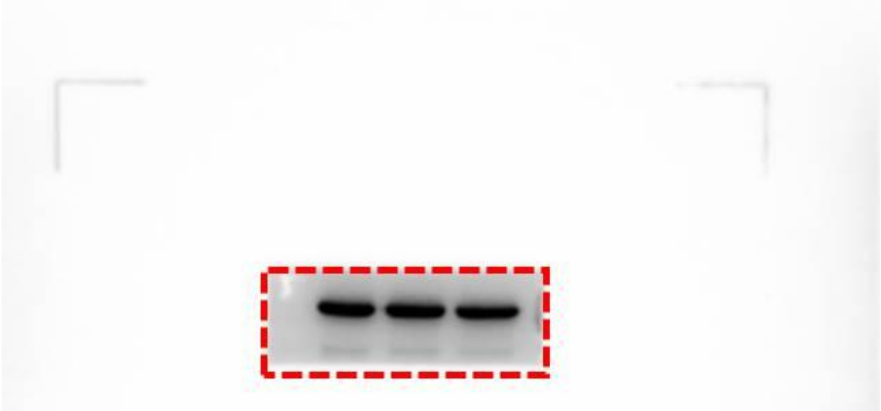

GAPDH

Figure 5D

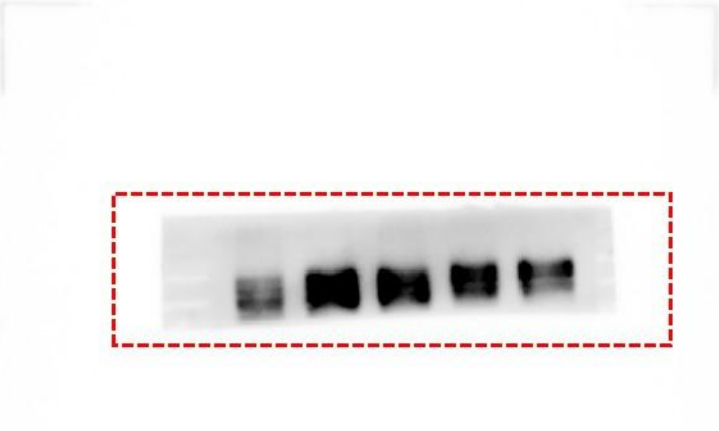

LAMP1

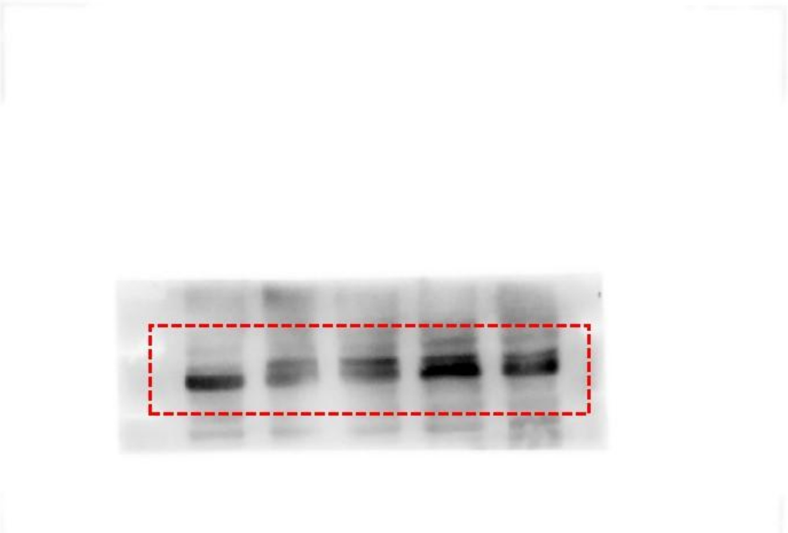

p62

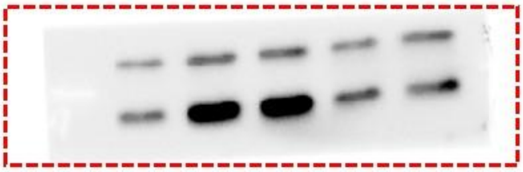

LC3

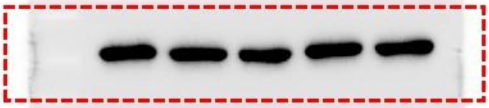

caspase 3

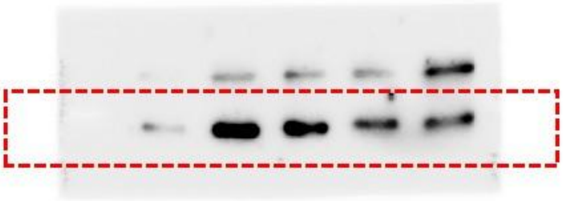

cleaved caspase 3

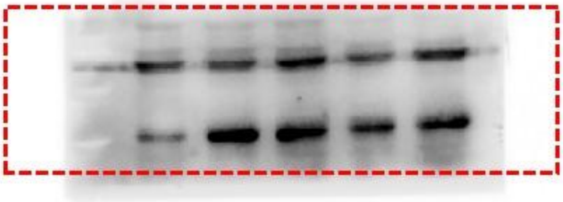

caspase 9 & cleaved caspase 9

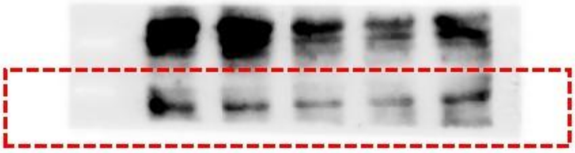

Bcl-2

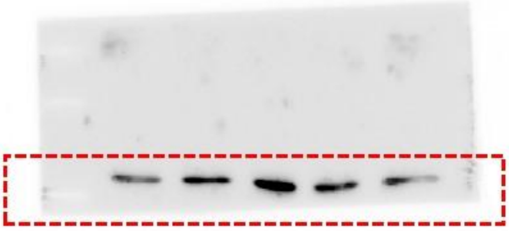

Bax

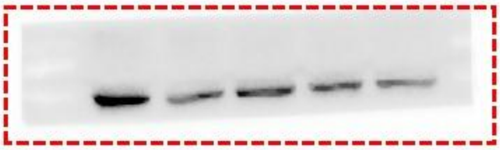

SHMT2

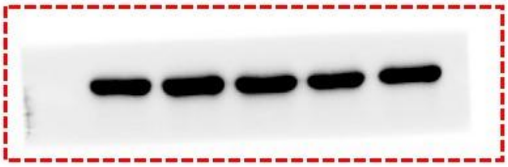

GAPDH

Figure 5G

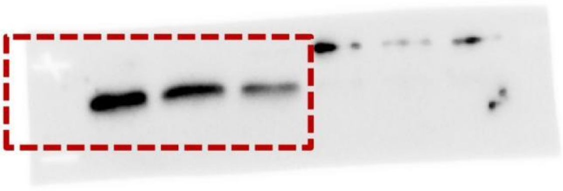

Mitochondria cytochrome C

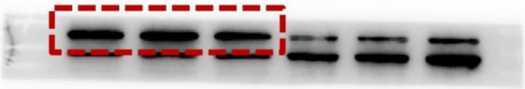

Hsp60

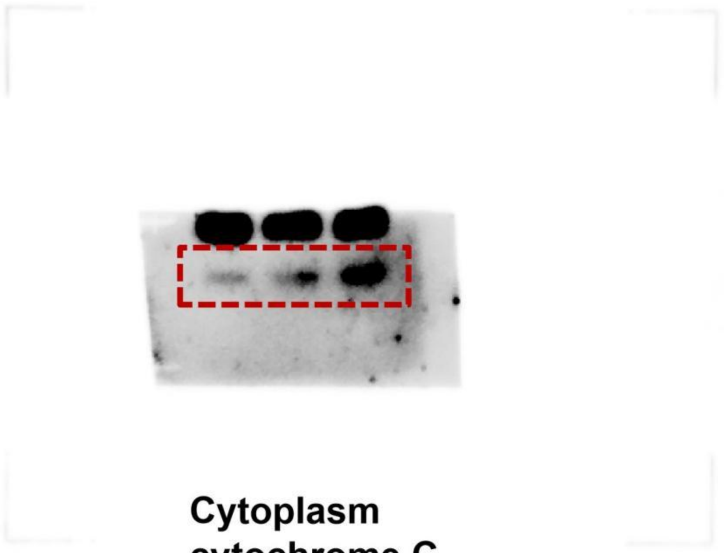

Cytoplasm
cytochrome C

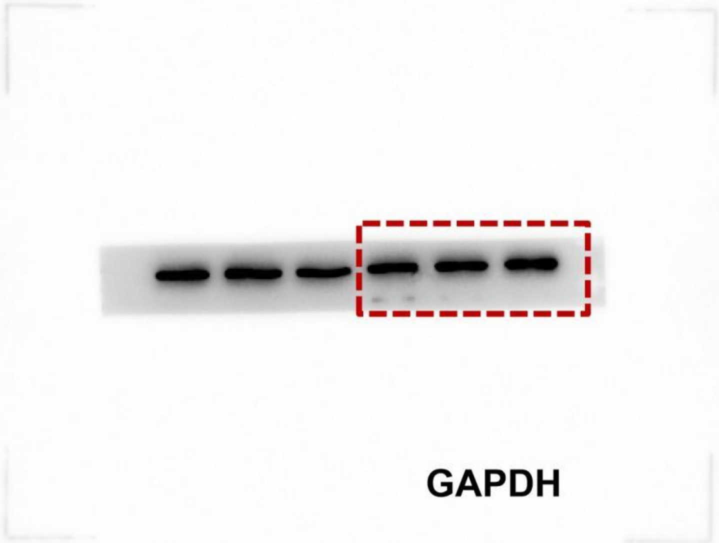

GAPDH

Figure S1A

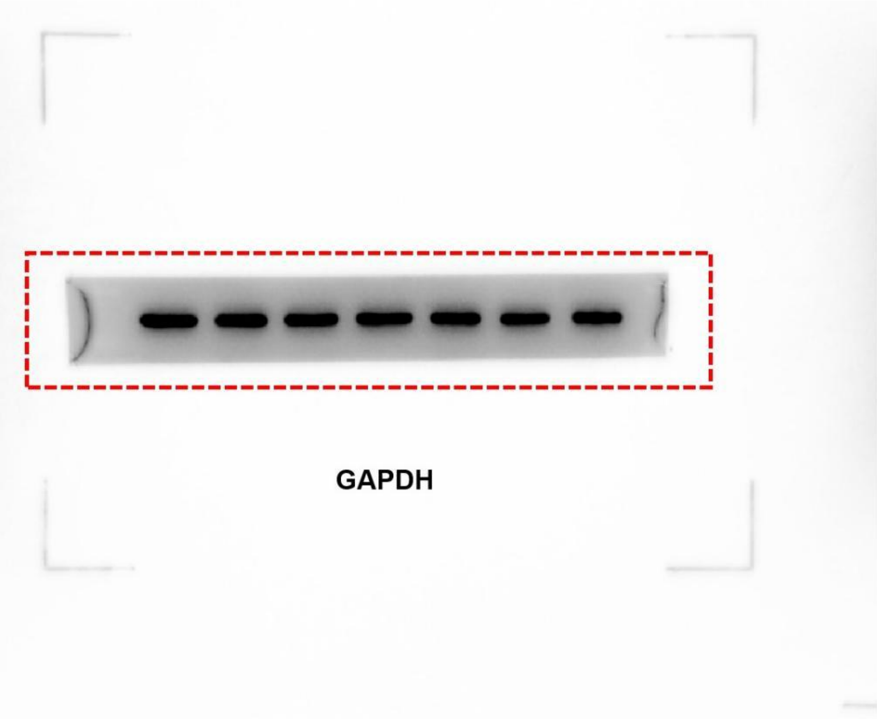

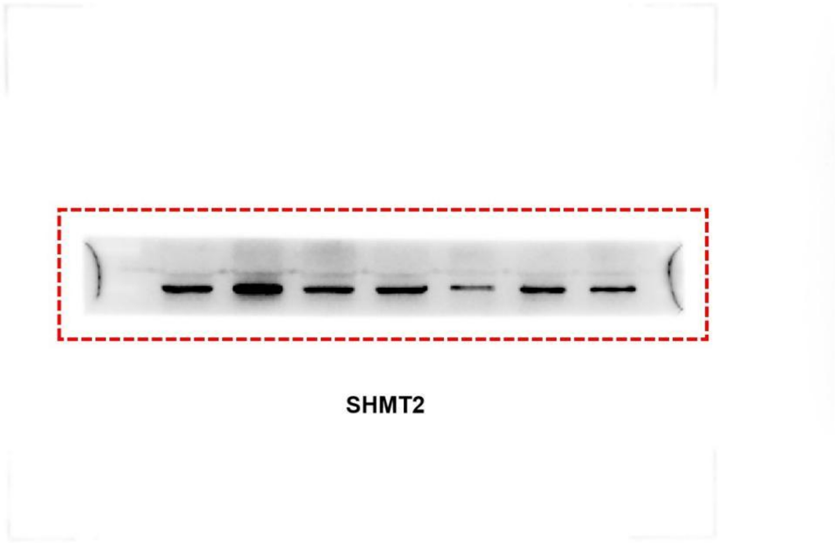

Figure S1B--ACHN

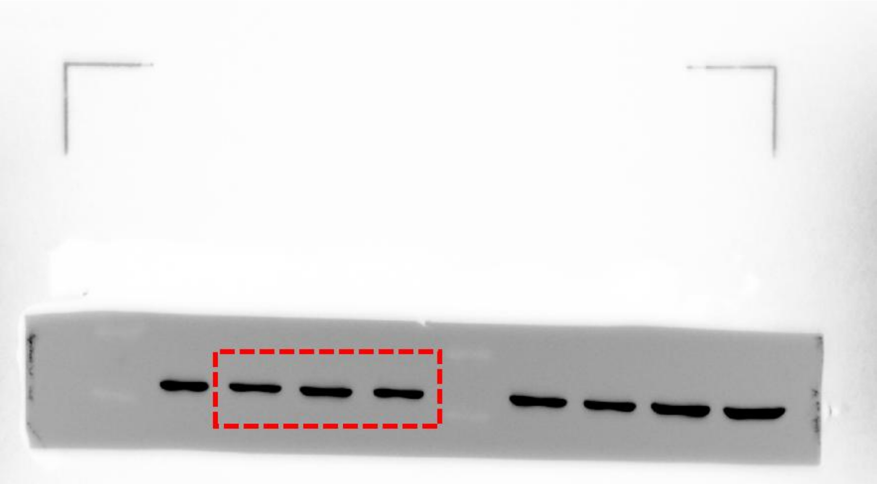

Actin

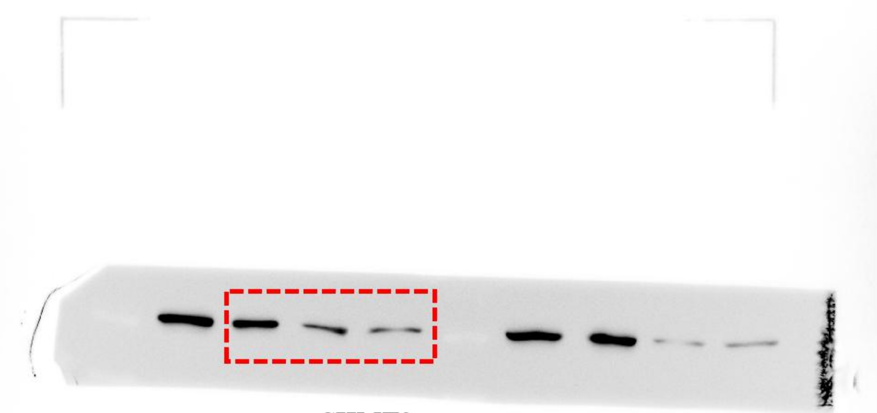

SHMT2

Figure S1B--A498

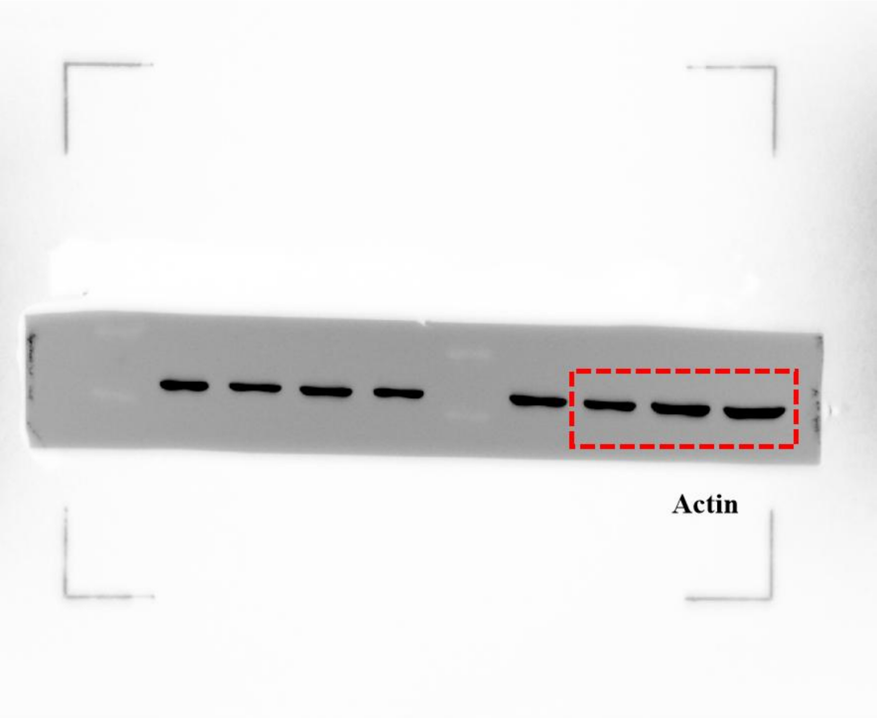

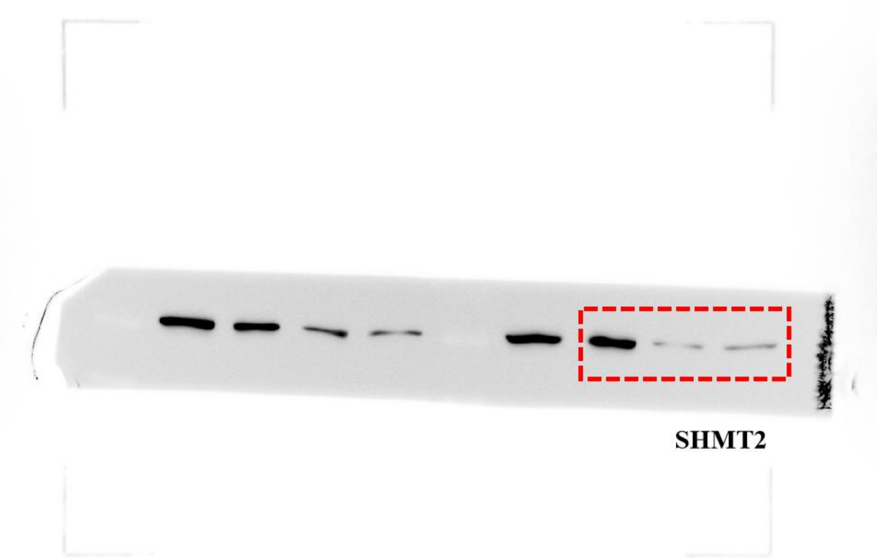

Figure S2- A498

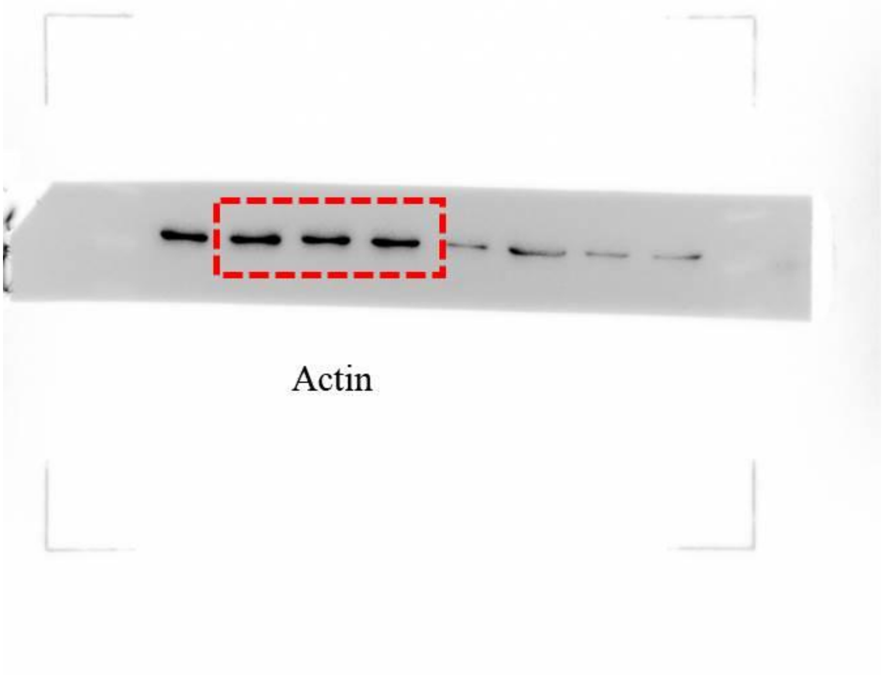

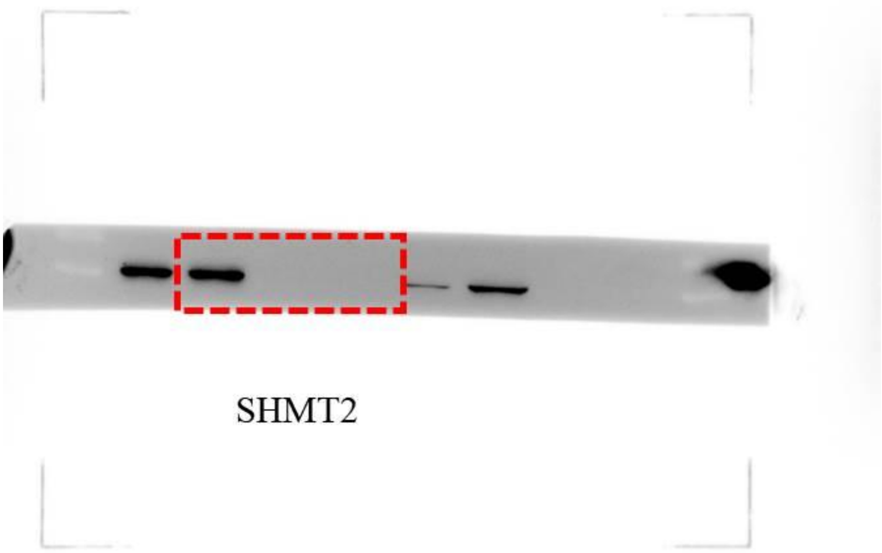

Figure S2- 786-O

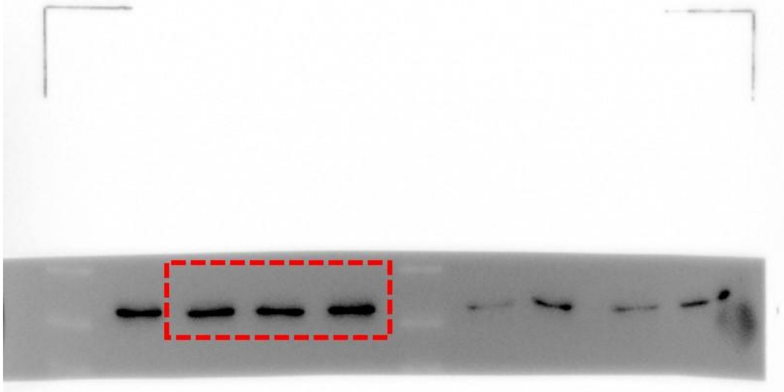

GAPDH

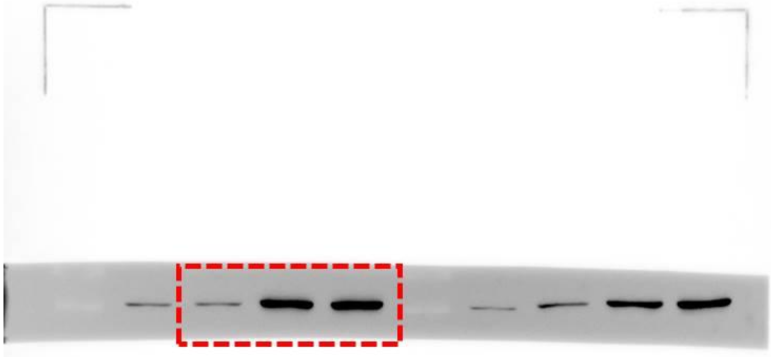

SHMT2

Figure S5A

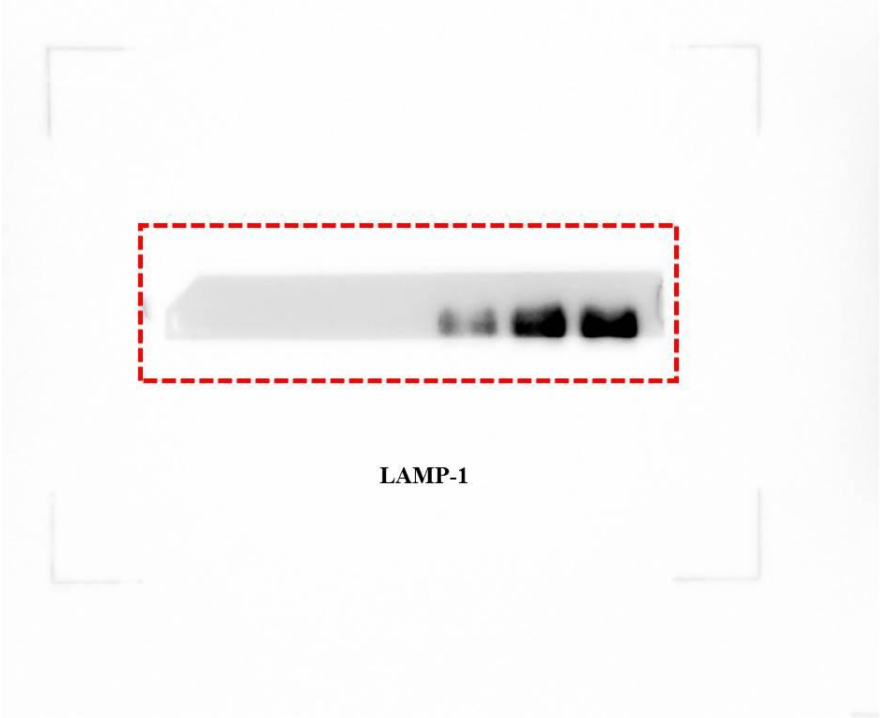

LAMP-1

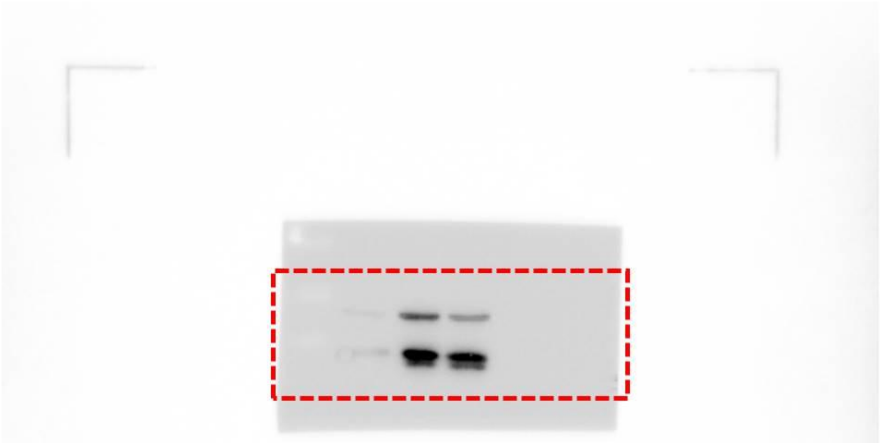

Cathepsin B

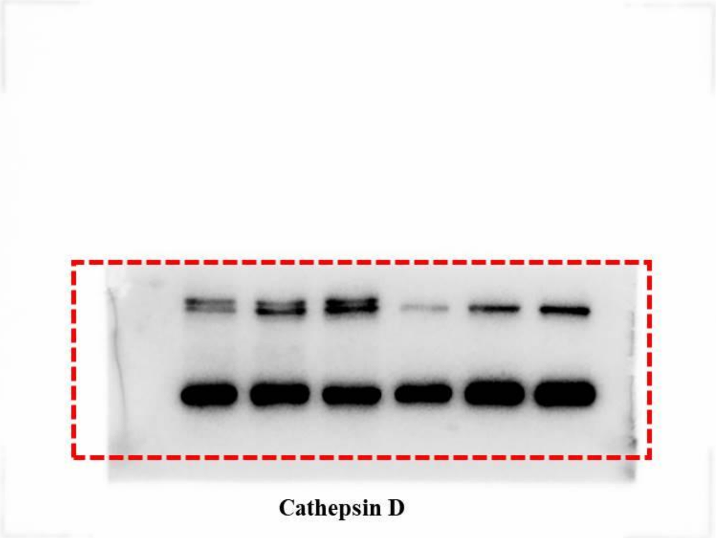

Cathepsin D

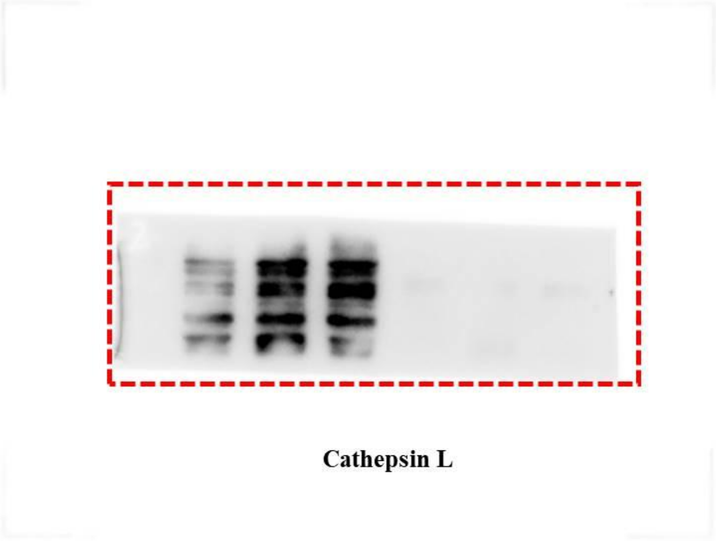

Cathepsin L

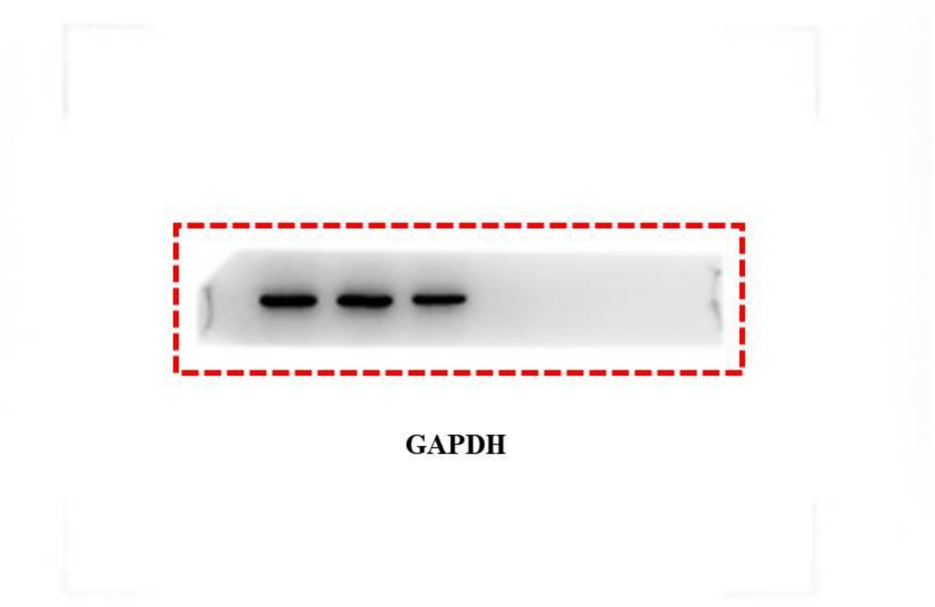

Figure S6E

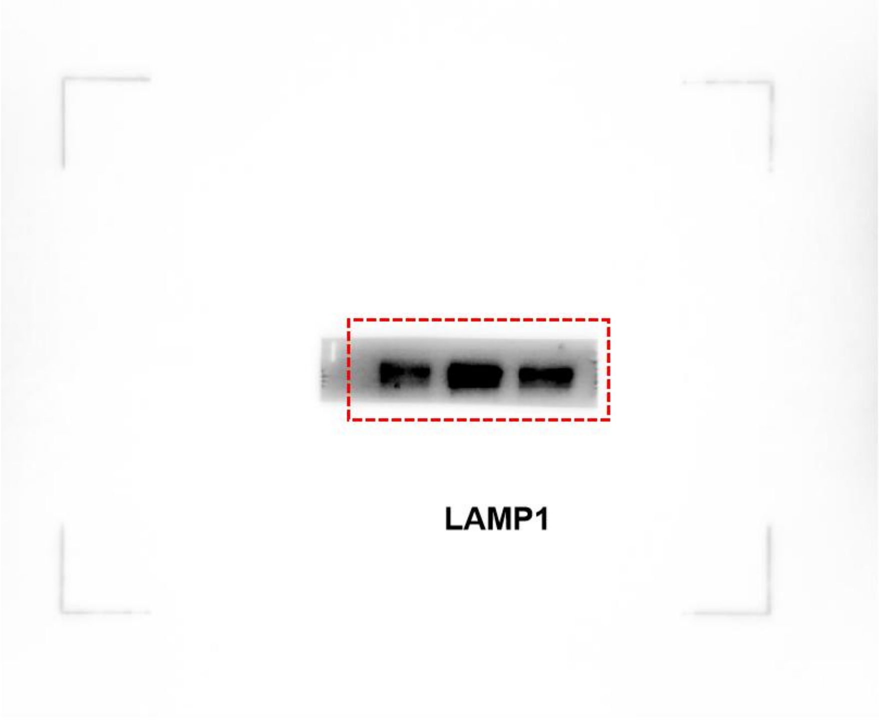

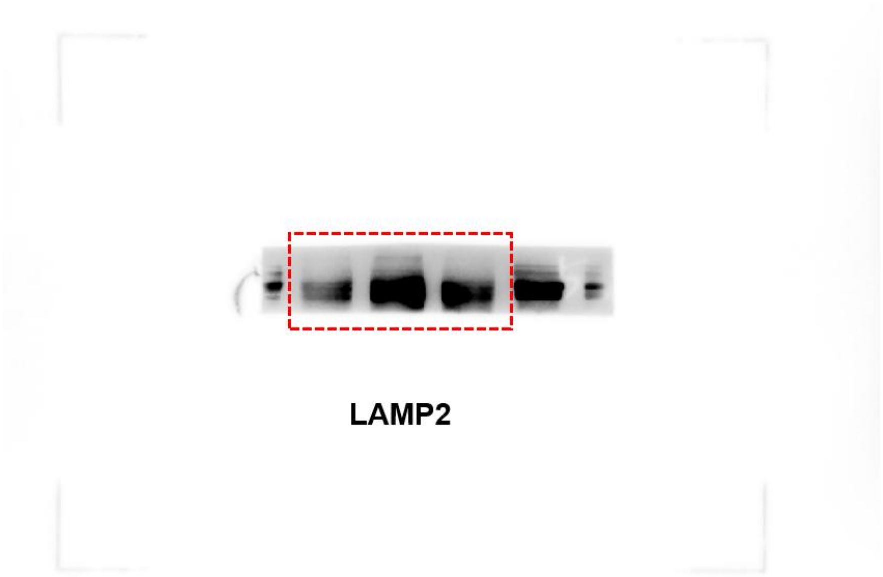

LAMP2

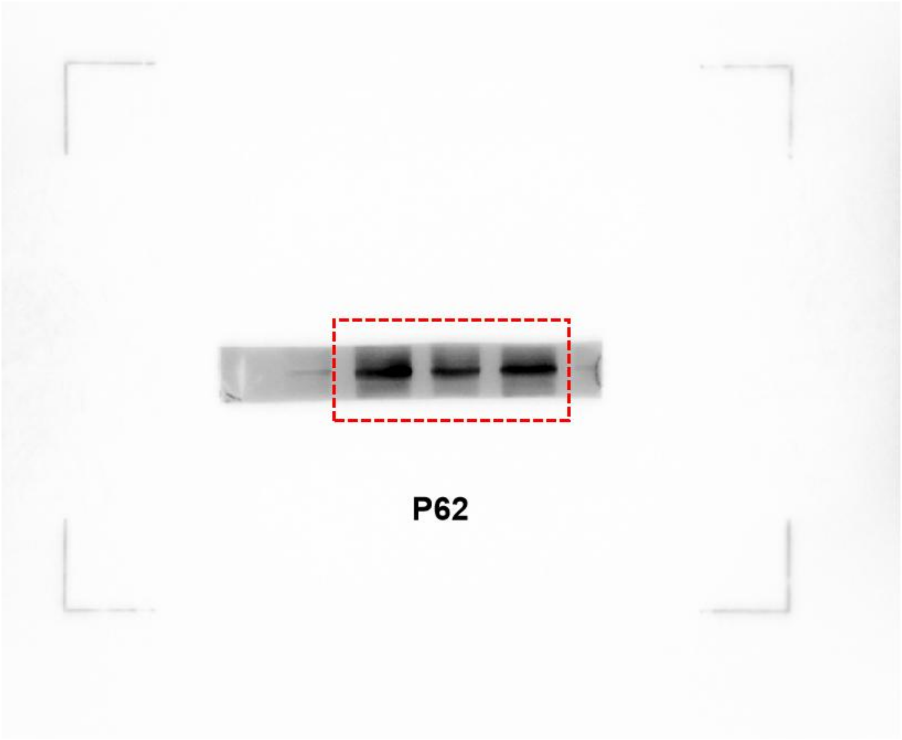

P62

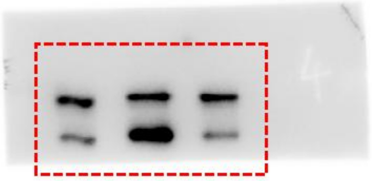

LC3

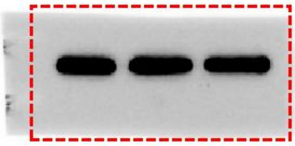

Caspase 3

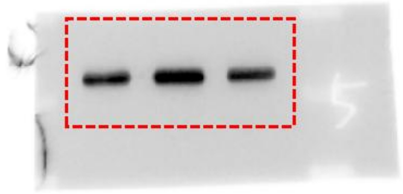

cleaved Caspase 3

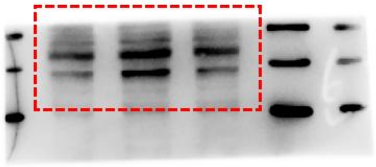

Caspase 9 & cleaved Caspase 9

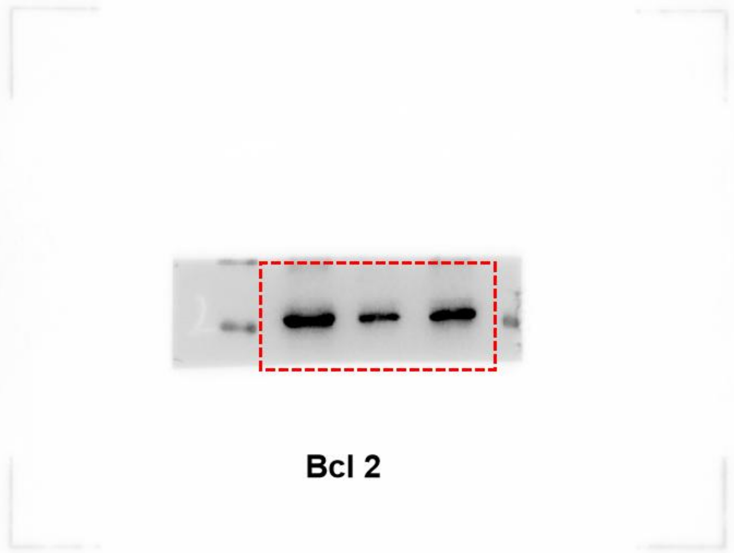

Bcl 2

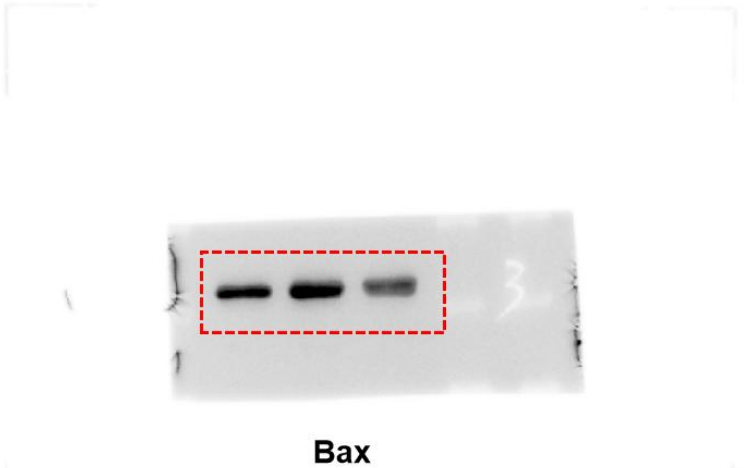

Bax

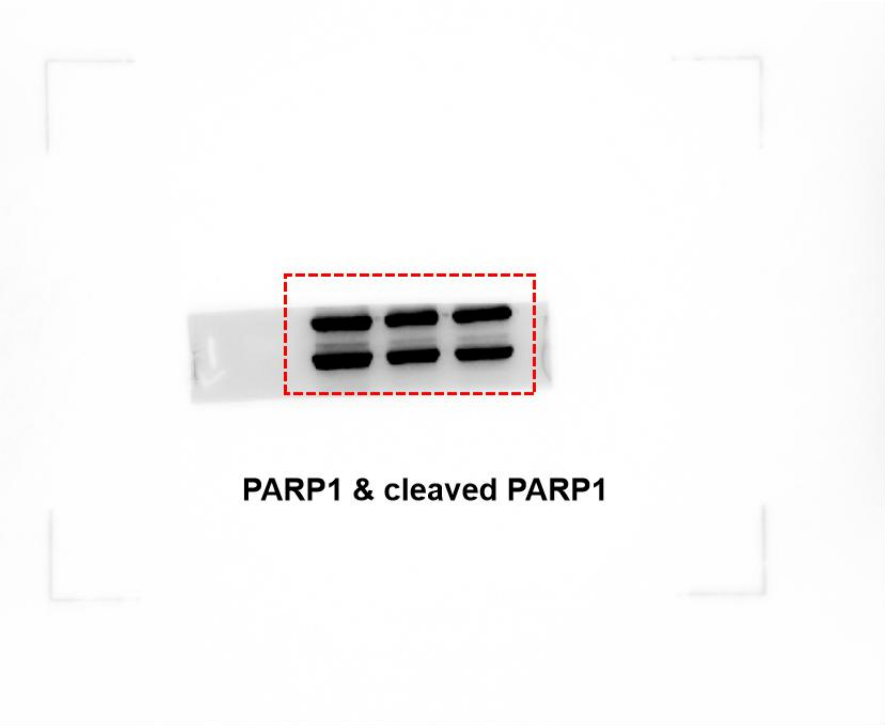

PARP1 & cleaved PARP1

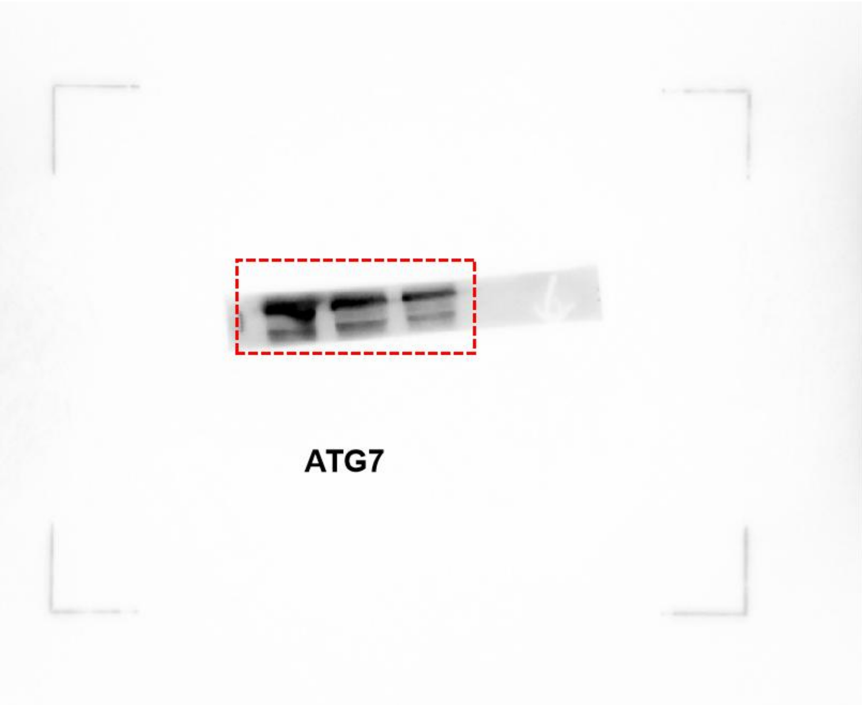

ATG7

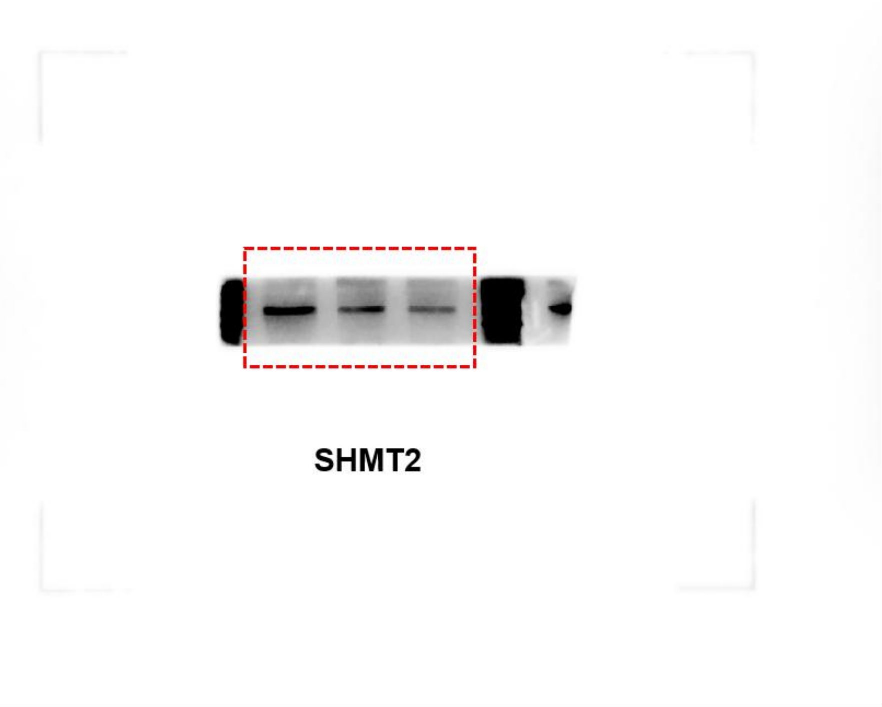
SHMT2

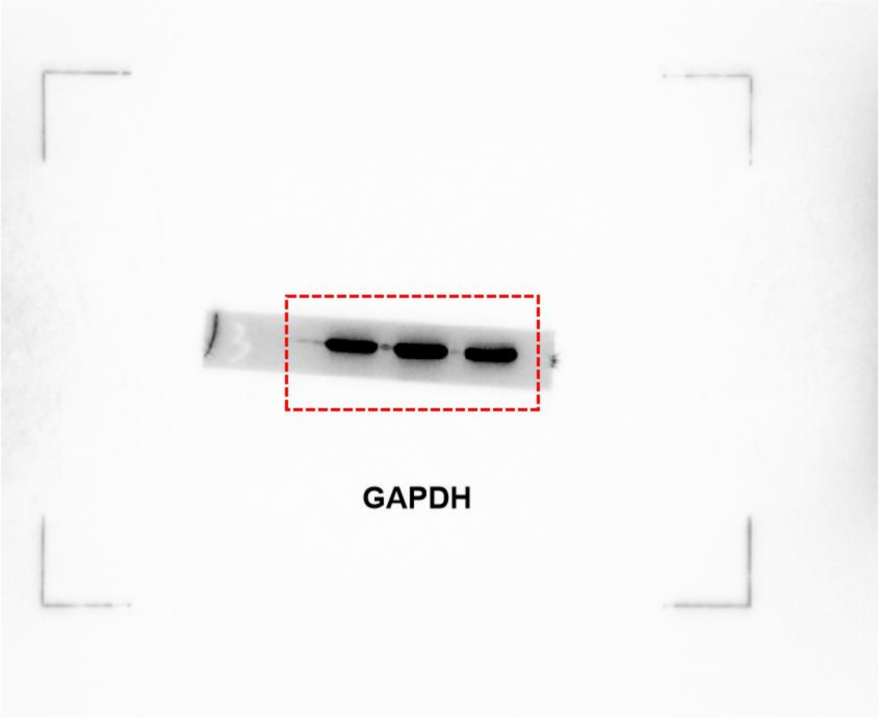
GAPDH

Figure S7B

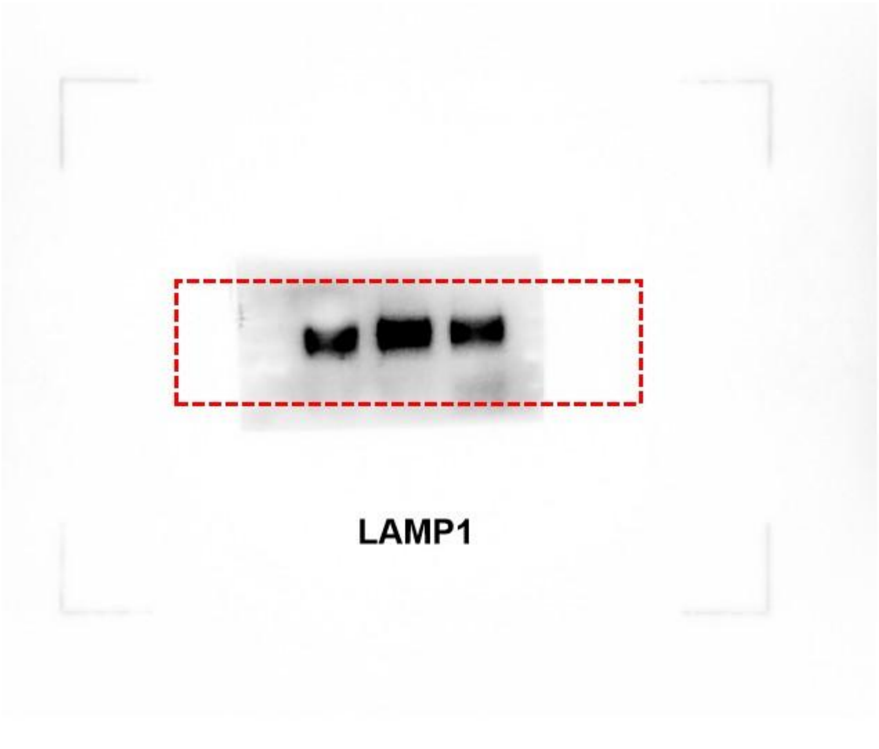

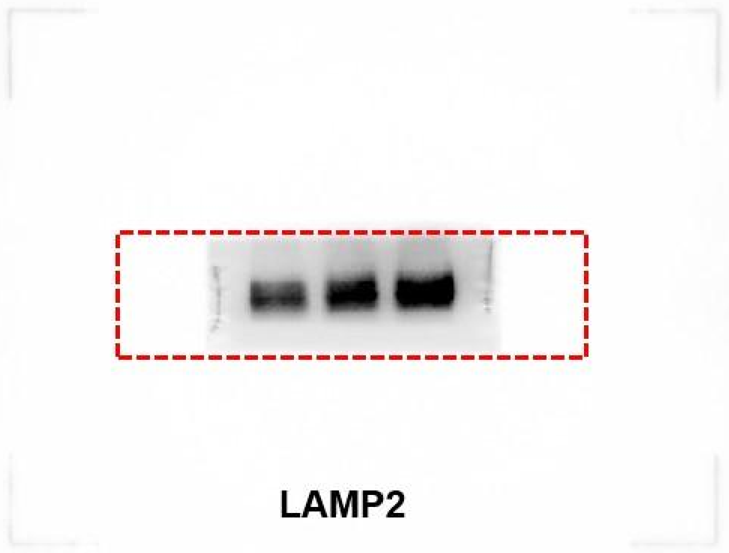

LAMP2

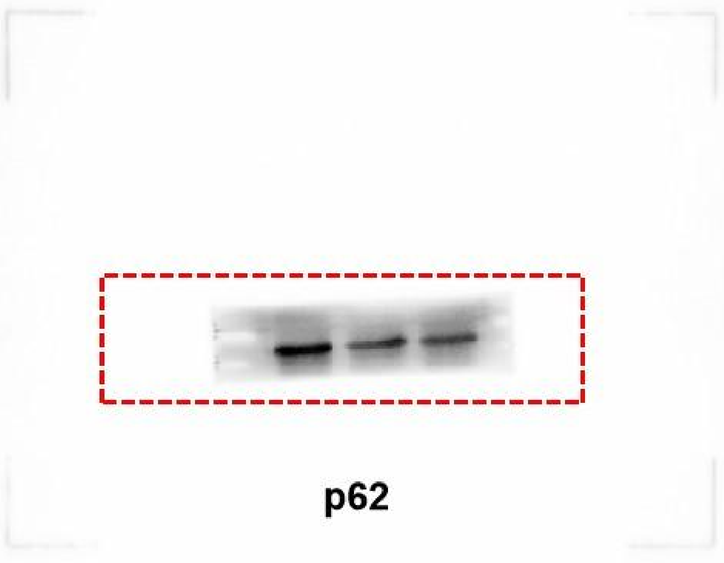

p62

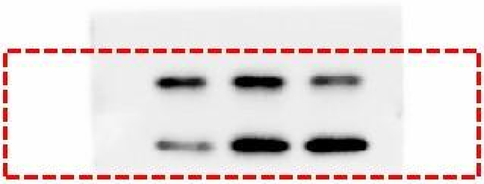

LC3

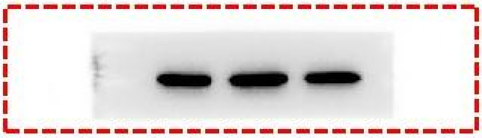

caspase 3

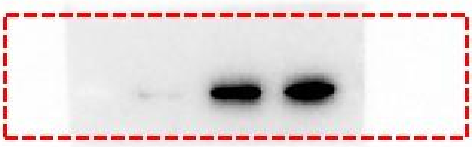

cleaved-caspase 3

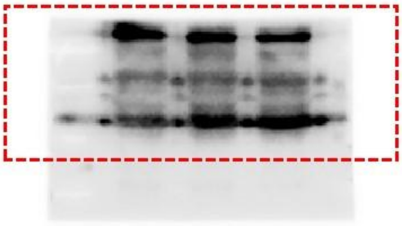

caspase 9 & cleaved caspase 9

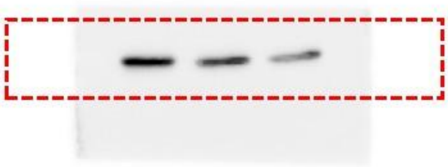

Bcl-2

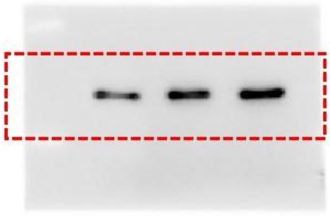

Bax

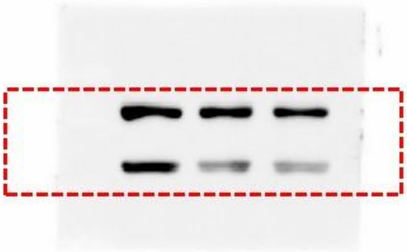

PARP-1 & cleaved PARP-1

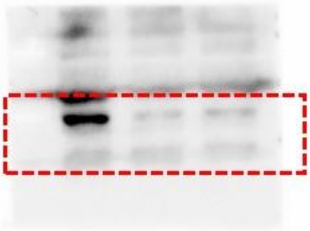

SHMT2

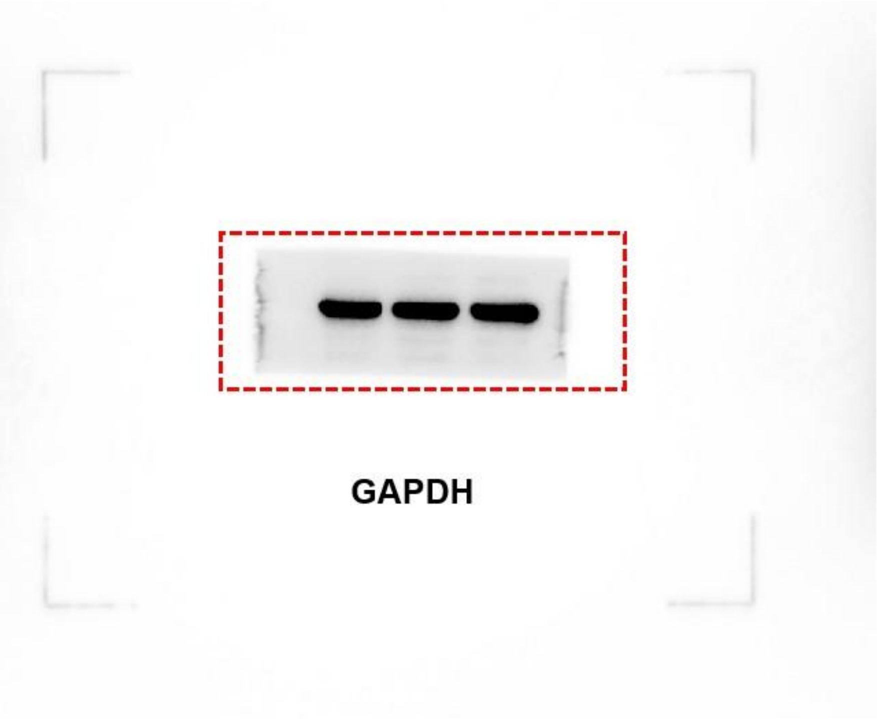

GAPDH
