# Supplementary material for: Serine hydroxymethyltransferase 2 knockdown induces apoptosis in ccRCC by causing lysosomal membrane permeabilization via metabolic reprogramming
Source: Cell Death Dis. 2023 Feb 20;14(2):144. doi: 10.1038/s41419-023-05677-4 (PMC9941282; doi:10.1038/s41419-023-05677-4)
Supplement: Supplementary file 4 — Certificate of English Language Editing [file 41419_2023_5677_MOESM4_ESM.pdf]

Certificate of English Language Editing

Manuscript Title:

Serine hydroxymethyltransferase 2 knockdown induces apoptosis in ccRCC by causing lysosomal membrane permeabilization via metabolic reprogramming

Date of Revision:

September 29, 2022

Abstract:

Serine hydroxymethyltransferase 2 (SHMT2) plays an important role in converting serine to glycine and supplying carbon to one-carbon metabolism to sustain cancer cell proliferation. However, the expression, function, and underlying mechanisms of SHMT2 in clear cell renal cell carcinoma (ccRCC) remain largely unknown. In this study, we demonstrated that SHMT2 was upregulated in ccRCC tissues compared with controls and associated with patient survival. SHMT2 knockdown inhibited proliferation, migration, and invasion in ccRCC cells. Overexpression of SHMT2 promoted tumor progression. Mechanistically, SHMT2 depletion disrupted one-carbon metabolism, increased reactive oxygen species levels, and decreased ATP levels via metabolic reprogramming, disrupting cell homeostasis. The SHMT2 knockdown-induced stress activated autophagy. A proportion of autophagosomes fused with lysosomes, resulting in lysosomal membrane permeabilization (LMP) and leakage of lysosomal contents into the cytoplasm, which eventually led to apoptosis. Our work reveals that SHMT2 functions as an oncogenic gene to promote...

This document certifies that the manuscript listed above was copy edited for English language by LetPub, with regard to grammar, punctuation, spelling, and clarity. All of our language editors are native English speakers with long-term experience in editing scientific and technical manuscripts. We are committed to leveling the playing field for researchers whose native language is not English.

- Documents receiving this certification should be regarded as having undergone professional editorial revision for English language before submission. However, the authors may accept or reject LetPub's suggestions and changes at their own discretion and LetPub does not have editorial control over the submitted documents.
- The language quality of the submitted document is the sole responsibility of the submitting authors subject to those authors' adherence to LetPub's revisions and instruction. LetPub's provision of service does not constitute a guarantee or endorsement of the authors' work herein.
- Neither the research content nor the authors' intended meaning were altered in any way during the editing process.
- If you have any questions or concerns about this edited document, please contact us at support@letpub.com

LetPub
Your Manuscript Editor

LetPub is an author service brand owned and operated by Accdon LLC. Headquartered in the Boston area, we are a full-spectrum author services company with a large team of US-based certified language and scientific editors, ISO 17001 accredited translators, and professional scientific illustrators and animators. We advocate ethical publication practices and are an official member of the Committee on Publication Ethics (COPE).

For more information about our company, services, and partnership programs, please visit www.letpub.com.

© 2022 Accdon, LLC. All Rights Reserved. Tel: 1-781-202-9968 Email: info@accdon.com Address: 400 Fifth Ave, Suite 530, Waltham, MA 02451, United States
